# The physics of bacterial decision making

**DOI:** 10.3389/fcimb.2014.00154

**Published:** 2014-10-30

**Authors:** Eshel Ben-Jacob, Mingyang Lu, Daniel Schultz, Jose' N. Onuchic

**Affiliations:** ^1^Center for Theoretical Biological Physics, Rice UniversityHouston, TX, USA; ^2^Department of Biosciences, Rice UniversityHouston, TX, USA; ^3^School of Physics and Astronomy and The Sagol School of Neuroscience, Tel-Aviv UniversityTel-Aviv, Israel; ^4^Department of Systems Biology, Harvard Medical SchoolBoston, MA, USA; ^5^Department of Physics and Astronomy, Rice UniversityHouston, TX, USA; ^6^Department of Chemistry, Rice UniversityHouston, TX, USA

**Keywords:** gene circuits, computational modeling, noise management, cell fate determination, sporulation and competence, *Bacillus subtilis*, cell communication

## Abstract

The choice that bacteria make between sporulation and competence when subjected to stress provides a prototypical example of collective cell fate determination that is stochastic on the individual cell level, yet predictable (deterministic) on the population level. This collective decision is performed by an elaborated gene network. Considerable effort has been devoted to simplify its complexity by taking physics approaches to untangle the basic functional modules that are integrated to form the complete network: (1) A stochastic switch whose transition probability is controlled by two order parameters—population density and internal/external stress. (2) An adaptable timer whose clock rate is normalized by the same two previous order parameters. (3) Sensing units which measure population density and external stress. (4) A communication module that exchanges information about the cells' internal stress levels. (5) An oscillating gate of the stochastic switch which is regulated by the timer. The unique circuit architecture of the gate allows special dynamics and noise management features. The gate opens a window of opportunity in time for competence transitions, during which the circuit generates oscillations that are translated into a chain of short intervals with high transition probability. In addition, the unique architecture of the gate allows filtering of external noise and robustness against variations in circuit parameters and internal noise. We illustrate that a physics approach can be very valuable in investigating the decision process and in identifying its general principles. We also show that both cell-cell variability and noise have important functional roles in the collectively controlled individual decisions.

## Introduction

Genetically identical cells are capable of stochastically differentiating into various phenotypes with unique attributes. This survival strategy allows a population to continuously assign specialized cells to deal with possible drastic changes in conditions (Kaern et al., 2005; Maamar and Dubnau, 2005; Süel et al., 2006, 2007; Maamar et al., 2007; Schultz et al., 2007, 2012, 2013; Acar et al., 2008; Losick and Desplan, 2008; Raj and van Oudenaarden, 2008; Be'er et al., 2009, 2011; Ben-Jacob and Schultz, 2010; Sirota-Madi et al., 2010). The stochastic differentiations into new phenotypes that determine the fate of the cell are coordinated by cell-cell communication, but still provide each cell with the flexibility to choose its own phenotype according to the particular conditions it encounters, while in harmony with other cells. Many different phenotypes communicate and contribute for the greater good of the colony by jointly performing a large variety of tasks (Aguilar et al., 2007; Lopez and Kolter, 2010). Coordinated individual decisions in a population of high cellular diversity require special mechanisms to guarantee that the individual decisions (transition probabilities) are regulated by the state of the population as a whole. As we will show, bacteria evolved intricate cell-cell communication that is utilized to execute the collective decisions. In addition, the decision circuits must have special capacity for noise management, allowing the bacterium to determine fate by “playing dice with controlled odds” (Ben-Jacob and Schultz, 2010). Cellular capacity to manage the odds entails both means to regulate and program the noise level and means to encode the effect of the noise on circuit performance (Maamar et al., 2007; Schultz et al., 2007, 2009; Süel et al., 2007; Acar et al., 2008; Losick and Desplan, 2008; Raj and van Oudenaarden, 2008). Several studies have illustrated that circuit architecture, i.e., the connectivity map of a circuit gene, can encode distinct noise behaviors critical to the function implemented by the circuit (Kollmann et al., 2005; Süel et al., 2006, 2007; Maamar et al., 2007; Cagatay et al., 2009; Kittisopikul and Süel, 2010).

The decision-making between sporulation and competence is a typical example of how genetic regulatory networks utilize noise for performance of cellular differentiation. Many bacterial strains (such as the in *Bacillus subtilis* used as the model species here), ultimately respond to severe environmental stress (such as starvation), by forming endospores—dormant cells that are extremely resistant to various hazards such as heat, radiation and toxic chemicals. The sporulation process involves an asymmetric division, and is followed by termination of metabolic activity in the daughter cell (i.e., the spore) and death by lysis of the mother cell. Sporulation is not initiated spontaneously upon nutrient limitation but kept as a last resort. Initially, cells employed many other tactics to survive the stress. When facing stress, *Bacillus subtilis* has been identified to differentiate into up to eight different phenotypes. These phenotypes include flagellated motile cells that seek for new food sources, cells that secrete hydrolytic enzymes to scavenge extracellular polysaccharides and proteins and also cannibal cells that feed on their peers (Aguilar et al., 2007; Lopez and Kolter, 2010). When other tactics fail in surviving the stress, sporulation is the fate chosen by a majority of the cells. Lysis of the sporulating cells releases material that is taken up by a small number of competent cells. During sporulation, the individual cells are able to switch into competence and can uptake from lysed cells the genetic material that is used as a food source, as material for DNA repair and even as new genetic information. The competent cells can switch back into vegetative growth after about one day, and proceed toward sporulation if still needed (Kuchina, 2011).

Bacterial decisions between sporulation and competence are carried on by complex gene network comprised of many genes interacted via complicated circuitry. We show that the network complexity can be considerably simplified by taking physics approach. This is done by reducing the complete integrated network into simpler functional modules, and then further reducing the modules into even simpler regulatory circuit motifs comprised of two or three genes. In the next section we present the dynamics of simple gene circuits and their associated computational principles (Novák and Tyson, 2008; Lu et al., 2013a). In the subsequent section we introduce how these circuits are interplayed when connected to form the elaborate gene network involved in bacterial decisions between sporulation and competence. We then show that it is possible to devise tractable models for the individual modules and utilize them to reveal the underlying general biological principles operative at the decision network to execute collective decisions.

We also show that studying the decision system brings new challenges from a physics perspective. For example, one of the key modules is a stochastic switch whose transition probability is regulated by the population density and population stress. From a physics perspective this is like a system composed of two-state elements with the transitions between the two states being coupled to two order parameters with long-range interaction. The stochastic switch is also regulated by a timer whose clock rate is determined according to the stress sensed by the individual cell, but is collectively normalized by the stress of the whole population. The situation becomes even more challenging since the clock rate is also regulated by the integration of stress at previous times.

The two modules controlling entrance into sporulation and competence are coupled by a decision gate with special architecture giving rise to oscillatory dynamics (Schultz et al., 2009, 2013). Each oscillation opens a short interval with high transition probability, “turning oscillations into opportunity spikes” (Schultz et al., 2013). We also explain that the gate has very special noise management capabilities (Schultz et al., 2013). It is important to note that while a physics approach can be very valuable in investigation of the decision process and identification of general principles, one has to keep in mind that unlike physical systems, biological systems evolved to perform tasks. Consequently some fundamental new principles are involved. For example, both cell-cell variability and noise have important functional roles in the collectively controlled individual decisions.

## Gene circuits dynamics and computational principles

The decision networks involved in cell fate determinations have a convoluted architecture of many interacting genes. Yet, the elaborated complexity of these signal transduction networks can be simplified, since their organization is that of simpler modules that are linked to each other, each with its own functional role. The modeling of the modules themselves can be further simplified, as they are comprised of regulatory motifs composed of just a few genes (Lu et al., 2013a).

**Figure 1:**
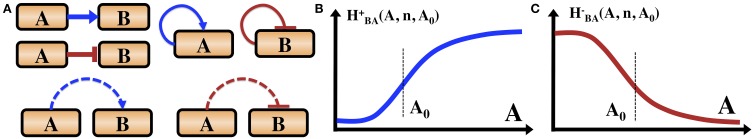
**Introduction of the gene circuit notations. (A)** Transcription and phosphorylation regulations. Blue arrows indicate transcription activation. Red bars indicate transcription inhibition. Dashed blue arrows indicate phosphorylation. Dashed red bars indicate dephosphorylation. We also illustrate that a gene can be self-activator or self-inhibitor. Some proteins can auto-phosphorylate (not shown here). **(B)** The functional form of an excitatory Hill function describing the action of an activator TF as is defined in the text. **(C)** The shape of an inhibitory Hill function describing the action of a repressor TF as is defined in the text.

The main forms of regulation mechanisms for these circuits fall into three categories: (1) *Transcriptional regulation*—proteins called transcription factors (TF), which regulate the rate of the gene transcription (expression) by binding to a specific segment of DNA termed promoter. The TFs can be either excitatory (activator) which increase the transcription rate, or inhibitory (repressor) which decrease the transcription rates. **(2)** *Translational regulation*—special genes (miRNA), encode for short segments of RNA which can bind to the mRNA of other genes and inhibit their translation into proteins. **(3)** *Post-translational modification*—many proteins are activated only by conformational changes after being modified, such as when bound to a phosphate group in a process called phosphorylation. Specialized proteins can phosphorylate or dephosphorylate other target proteins, and in some cases can also auto-phosphorylate. In Figure 1 we introduce the symbols adopted in this article to indicate the transcription regulations and phosphorylation regulation. We do not include translational regulation, since it is not part of the example of sporulation vs. competence decision network discussed in this article.

### Transcriptional regulation

The most fundamental regulatory process is transcription regulation. The same gene can regulate the transcription of various genes and different genes can regulate the transcription of a given gene, leading to a transcriptional regulatory network. The typical equations describing transcription activation and inhibition of gene B by gene A are given by Equations (1) and (2) respectively.

(1) $$\text{dB/dt}={\text{g}}_{\text{B0}}+{\text{g}}_{\text{BA}}{\text{H}}_{\text{BA}}^{+}(\text{A},\text{n},{\text{A}}_{0})-{\text{k}}_{\text{B}}\text{B}$$

(2) $$\text{dB/dt}={\text{g}}_{\text{B0}}+{\text{g}}_{\text{BA}}{\text{H}}_{\text{BA}}^{-}(\text{A},\text{ n},\;{\text{A}}_{0})-{\text{k}}_{\text{B}}\text{B},$$

where g_B0_ is the basal transcription rate of gene B, k_B_ is the spontaneous degradation rate of protein B and H^+^*_BA_* (A, n, A_0_) and H^−^_BA_ (A, n, A_0_) are the excitatory and inhibitory Hill functions whose functional form are given by:

(3) $${\text{H}}_{\text{BA}}^{+}(\text{A},\text{ n},\;{\text{A}}_{\text{0}})\;\equiv {\left(\text{A}\right)}^{\text{n}}\text{/}[{\left({\text{A}}_{\text{0}}\right)}^{\text{n}}+{\left(\text{A}\right)}^{\text{n}}]$$

(4) $${\text{H}}_{\text{BA}}^{-}(\text{A},\text{ n},\;{\text{A}}_{\text{0}})\;\equiv {\left({\text{A}}_{\text{0}}\right)}^{\text{n}}\text{/}[{\left({\text{A}}_{\text{0}}\right)}^{\text{n}}+{\left(\text{A}\right)}^{\text{n}}]$$

where n is the rank of the Hill function (non-linearity or cooperativity) and A_0_ is the midpoint concentration.

### Regulation by phosphorylation

Below we describe the mechanism of regulation of a protein B by phosphorylation. B is phosphorylated by a kinase A and dephosphorylated by a phosphatase C. The dynamics of B and B^*^ (phosphorylated B) are modeled by

(5) $$\text{dB/dt}={\text{g}}_{\text{B}}-{\text{p}}_{\text{A}}\text{AB + }{\text{d}}_{\text{C}}{\text{CB}}^{*}-{\text{k}}_{\text{B}}\text{B}$$

(6) $${\text{dB}}^{*}\text{/dt}={\text{p}}_{\text{A}}\text{AB}-{\text{d}}_{\text{C}}{\text{CB}}^{*}-{\text{k}}_{\text{B}}^{*}{\text{B}}^{*},$$

where p_A_ is the phosphorylation rate of B by A and d_C_ is the dephosphorylation rate of B^*^ by C. From a physics perspective, regulation by phosphorylation is a much faster process than transcriptional regulation. Before the concentration of a protein being transcriptionally regulated can feel the effects of changes in the binding state of its promoter, it takes tens of minutes for transcription of the genetic code into pre-mRNA, editing of the pre-mRNA into mRNA, migration of the mRNA from the nucleus (in eukaryotes) and translation into proteins. The timescale of phosphorylation events is much shorter, in the order of seconds or minutes. It is mainly determined by the time for diffusion and colocalization of the two proteins, since the exchange of phosphate is fast.

### Translational regulation

Translational regulation by miRNA plays a crucial role in eukaryotic genetic regulation. In general, miRNA can inhibit the production of the target protein by either degrading mRNA or inhibiting translation. Compared to the equations for transcriptional regulation, those for translational regulation are more complex as they involve dynamical equations for the miRNA, mRNA and the proteins. Since translation regulation is not part of the example of sporulation vs. competence decision network discussed in this article, we do not present it here and direct the interested reader to Ref (Ray et al., 2011; Lu et al., 2013a).

### Examples of simple gene circuits

**Figure 2:**
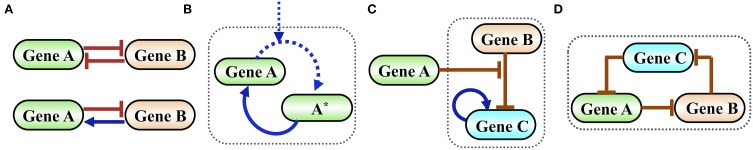
**Examples of simple functional gene circuits. (A)** Examples of two-gene circuits. Top is a classical toggle switch comprised of two mutually inhibiting genes described in details in the next section. Bottom is a classical flip-flop element comprised of two genes that are mutually inhibiting in one direction and activating in the other. **(B)** Example of a self-activating timer. Time is measured by the level of the phosphorylated protein A^*^, which is accumulated by external signal that phosphorylates A. In typical timers the gene A is self-activated by A^*^. **(C)** Example of an inhibition gated switch. Gene B inhibits the self-activating gene C from making transition into high expression (high level state). When the level of protein A increases it inhibits the inhibition of C by B, thus permits a stochastic transition into a state of high C. **(D)** Example of an oscillator (termed in systems biology as a repressilator) comprised of an inhibition loop among three genes. The origin of the oscillation is as follows: when the level of A increases, it inhibits B. As a result, the level of C increases leading to a decrease in the level of A and so on.

In Figure 2 we show examples of functional gene circuits (regulatory motifs) comprised of only a few interacting genes: the toggle switch and the flip-flop circuits (Figure 2A), the self-activating timer (Figure 2B), a gated switch (Figure 2C) and an oscillator (Figure 2D).

### Dynamical system approach to gene circuits

Studying genetic circuits by a dynamical systems approach usually involves the following elements: analysis of fixed points and their stability in the phase space, evaluation of the corresponding bifurcation diagrams and investigation of noise effects and transitions between possible states. The approach is illustrated here via the classical toggle switch (Figure 2A), a simple circuit that already leads to interesting dynamics, described by the following deterministic equations:

(7) $$\text{dA/dt}={\text{g}}_{\text{A0}}\text{ + }{\text{g}}_{\text{A}}{\text{H}}_{\text{AB}}^{-}(\text{B},\;{\text{n}}_{\text{B}},\;{\text{B}}_{\text{0}})-{\text{k}}_{\text{A}}\text{A}$$

(8) $$\text{dB/dt}={\text{g}}_{\text{B0}}\text{ + }{\text{g}}_{\text{B}}{\text{H}}_{\text{BA}}^{-}(\text{A},\;{\text{n}}_{\text{A}},\;{\text{A}}_{\text{0}})-{\text{k}}_{\text{B}}\text{B}$$

The circuit has two stable states, which can be described as logical states (0,1) and (1,0)—low level of A and high level of B, and vice versa. A more intricate variant is the asymmetric self-activating toggle switch (SATS) in which one of the genes (say gene B) is a self-activator. When the element is driven by an external signal I acting as a transcription factor of A, the dynamics is modeled by

(9) $$\text{dA/dt}={\text{G}}_{\text{A}}(\text{I},\text{ B})-{\text{k}}_{\text{A}}\text{A}$$

(10) $$\text{dB/dt}={\text{G}}_{\text{B}}(\text{B},\text{ A})-{\text{k}}_{\text{B}}\text{B},$$

where G_X_(A,B) is given by:

(11) $$\begin{array}{l} {\text{G}}_{\text{X}}(\text{A},\text{ B})\text{ = }({\text{g}}_{\text{X},\text{0}}-{\text{g}}_{\text{X},\text{A}}-{\text{g}}_{\text{X},\text{B}}\text{ + }{\text{g}}_{\text{X},\text{AB}}){\text{H}}^{-}(\text{A},\;{\text{n}}_{\text{A}},\;{\text{A}}_{\text{0}})\\ \;{\text{H}}^{-}(\text{B},\;{\text{n}}_{\text{B}},\;{\text{B}}_{\text{0}})\text{ + }({\text{g}}_{\text{X},\text{B}}-{\text{g}}_{\text{X},\text{AB}}){\text{H}}^{-}(\text{A},\;{\text{n}}_{\text{A}},\;{\text{A}}_{\text{0}})\\ \;+\;({\text{g}}_{\text{X},\text{A}}-{\text{g}}_{\text{X},\text{AB}}){\text{H}}^{-}(\text{B},\;{\text{n}}_{\text{B}},\;{\text{B}}_{\text{0}}){\text{+g}}_{\text{X},\text{AB}}. \end{array}$$

Note that this is a general result, gene X can be one of the genes A or B or the input signal I. g_*X*,0_, g_*X,A*_, g_*X,B*_ and g_*X,AB*_ are the individual transcription rates when the promoter of the gene X is in the free from, A-bounded form, B-bounded form and AB-bounded form respectively. Equation (11) was derived from the generic equation for the case of transcription regulation by two TFs A and B when both TFs bind to the promoter of the gene B at two different binding sites (Lu et al., 2013a).

**Figure 3:**
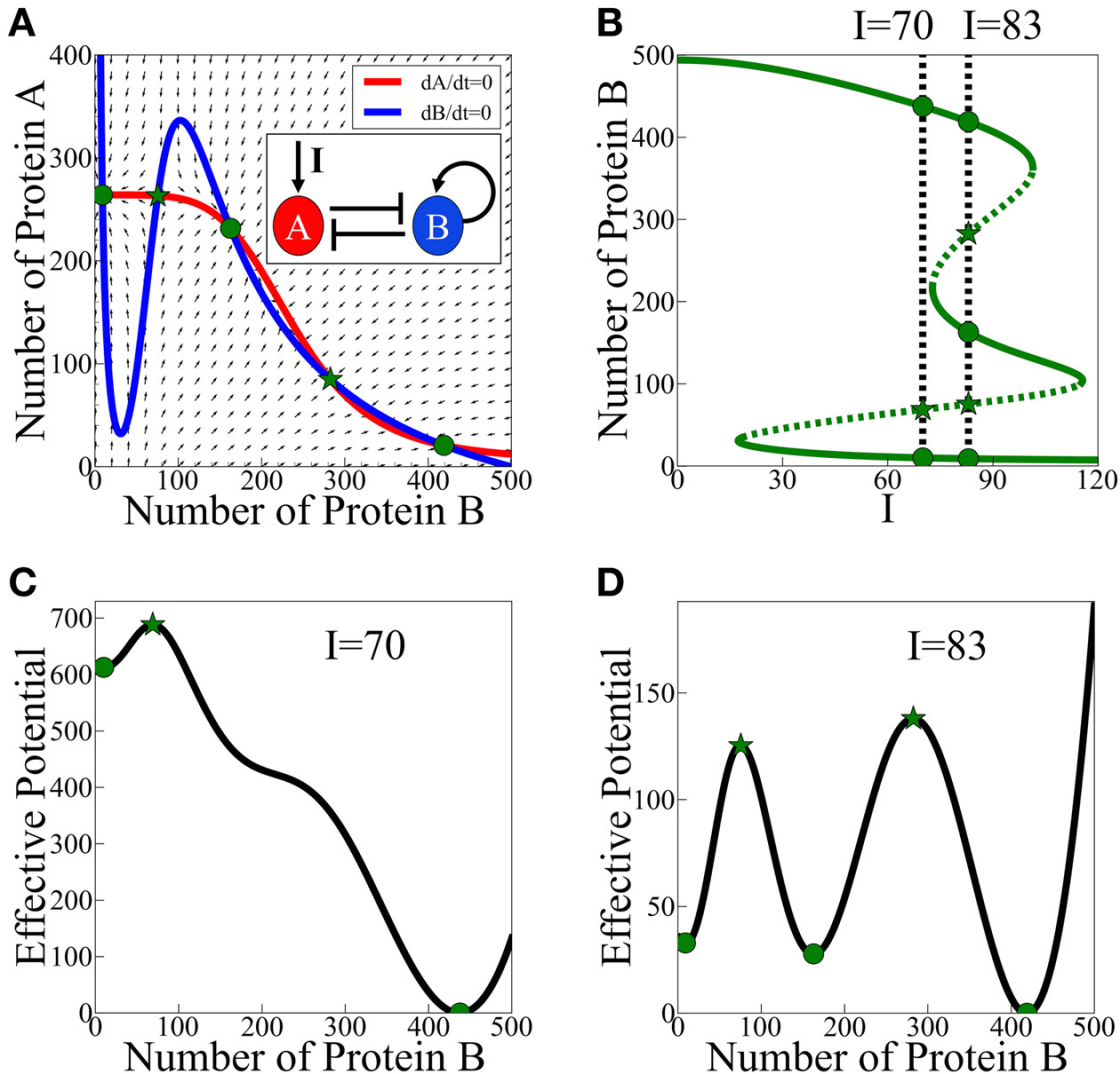
**Asymmetric SATS driven by an external signal I**. The results shown in this figure were computed, for the schematic circuit depicted in **(A)**, from Equations (9) and (10). **(A)** Phase-space showing the nullclines for a specific value of the input signal I when three meta-stable states coexist. **(B)** The bifurcation diagram as function of the input signal. The green solid lines represent the stable fixed points, while the green dotted lines represent the unstable fixed points. The two specified cases: two meta-stable states (the bistability on the left, *I* = 70), and three coexisting meta-stable states (tristability on the right, *I* = 83). Panels **(C,D)** show a 1-dimensonal effective potential computed along the *dA*/*dt* = 0 nullcline for *I* = 70 and *I* = 83 respectively. Green circles mark the stable fixed points and the green stars mark unstable saddle fixed points (Adopted from Lu et al., 2013a).

According to the analysis of the model, asymmetric SATS can have three coexisting meta-stable states for some specific parameters and input signal. The corresponding phase space for a given input signal is illustrated in Figure 3A and the bifurcation diagram as function of the input signal is presented in Figure 3B. In Figures 3C,D we show two examples (for two values of the input signal) of the 1-dimensional effective potential obtained by integrating the equations along the dA/dt = 0 nullcline. Here, we assume that A reaches to steady states much faster than B. The common analysis for the effective potential will be explained in detail in Section The Stochastic Switch.

### Integrated gene circuits

Simple regulatory motifs do not necessarily have a stand-alone function, but act as core elements integrated within functional modules. These modules are further integrated to form task-performing large-scale gene circuits such as the bacterial sporulation-competence decision network (Schultz et al., 2009) studied here. These decision systems (e.g., the meiosis regulatory network in yeast, Nachman et al., 2007, and the epithelial-mesenchymal decision networks in embryonic and cancer cells, Ben-Jacob et al., 2012) typically contain decision modules which are integrated with sensing and communication units. These units process information from various input signals and regulate the operation of the decision module. The sensing units detect information about the environment and the communication units exchange information between different cells to coordinate individual decisions.

Generally speaking, there are four main types of decision modules: (1) Inhibition gated stochastic switches like the ComK-ComS stochastic switch studied here. (2) Toggle switch based modules that are comprised of coupled self-activating toggle switches (SATS). These modules are common in the regulation of cell fate determination during embryonic development and tumorigenesis. (3) Feed-Forward-Loop (FFL) based modules that are comprised of coupled FFLs (some of which have also a backward coupling, FFBL, so they can have multiple states). (4) Mixed modules which are comprised of coupled SATS and FFLs.

### Gene circuits' architecture, noise managements and task performance

Although each decision module type has specific circuit architecture, they can all share similar features—transitions between coexisting two or more meta-stable states. This poses the fundamental question as to why a gene circuit of particular architecture is selected to execute a function that could, in principle, be performed by alternative architectures. Recent studies began to focus on the fundamental issue of Architecture-Noise-Performance relations of gene circuits in the context of Feed-Forward-Loop (FFL) motifs (Acar et al., 2008; Losick and Desplan, 2008) and showed that circuits that give rise to similar deterministic dynamics using different architecture have very different response to noise. Consequently it has been proposed that the Architecture-Noise-Performance relations may have driven evolutionary selection of the gene circuit architecture according to the desired effect of noise (e.g., rare vs. frequent transitions).

With regard to decision modules, it means that different architectures will give rise to different noise management characteristic and hence different transition rates and different functional dependence of the transition rates of the input signals. We propose that the specific architecture of the sporulation-competence decision network has been selected to guarantee that the decision process of the individual cells will be highly coordinated and executed within similar time windows, in a way that only a small fraction (about 5–10% of the cells) will make the transition into competence.

## Global view of the sporulation-competence decision-making system

**Figure 4:**
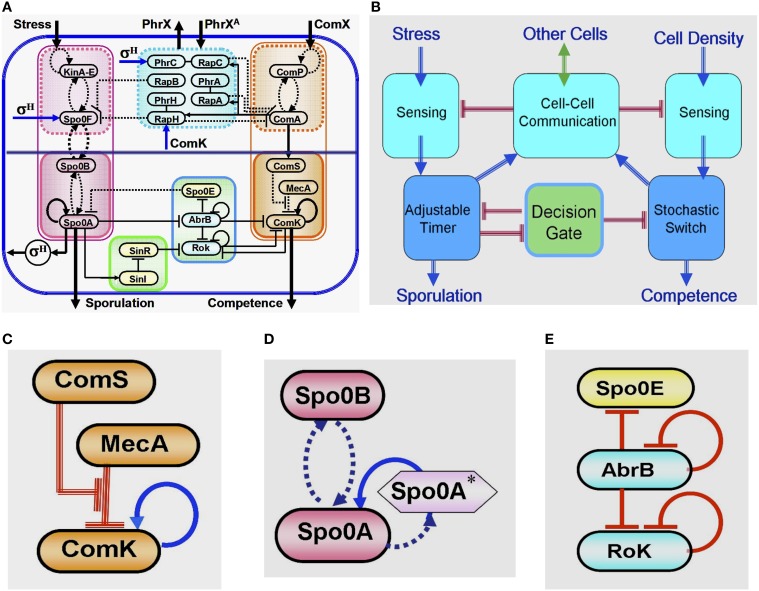
**Global presentation of the sporulation/competence decision system. (A)** Representation of the complete network. **(B)** Schematic representation showing the 5 modules that constitute the network. **(C)** This three-elements switch is comprised of: 1. a self-activator gene (ComK) whose value determines the transitions—the cell enters into competence above threshold level of ComK (this is why it is called competence master regulator). 2. A regulator gene (ComS) whose level is determined by input from the sensing and communication units. 3. A degrader complex (MecA) which degrades both the ComK protein and the ComS peptide in a competitive manner. The red parallel lines indicate regulation by degradation. **(D)** This two-element timer is comprised of: 1. the sporulation master regulator gene (Spo0A) which is self-activated by Spo0A^*^ (the phosphorylated Spo0A) (Novák and Tyson, 2008; Kuchina, 2011). Once the level of Spo0A^*^ exceeds a threshold value, the cell commits to sporulation (the sporulation process begins and cannot be reversed). This is why Spo0A is called the sporulation master regulator. In addition to Spo0A, the adaptable timer is comprised of a regulator Spo0B which regulates the clock rate—the rate of accumulation of Spo0A^*^—according to the rate its protein is phosphorylated by input from the stress sensing unit and the communication unit. When the level of Spo0B^*^ is decreased (the stress is lifted), Spo0A^*^ can phosphorylate Spo0B. This process leads to decrease in the level of Spo0A^*^ meaning reversing the timer. **(E)** This three-element gate allows transition into competence only within a “window of opportunity” between two values of Spo0A^*^. The special architecture of the circuit leads to generation of oscillatory behavior within the window of opportunity. As is shown in details further below (see Section The Decision Gate), within each oscillation the gate opens for a short time during which the inhibition of the stochastic switch is lifted.

Years of intensive experimental studies identified the tens of key regulatory genes and measured the associated physiological parameters that are involved in the sporulation-competence decision process of domesticated *B. subtilis*. Considerable effort has been devoted to simplify the complexity of this elaborated network (Figure 4A) by untangling the basic functional modules that are integrated to form the complete network (Schultz et al., 2009, 2013). It is now realized that the key modules are (Figure 4B): (1) a stochastic switch whose transition probability is normalized by signals from other cells; (2) an adaptable timer whose clock rate is normalized by the cell stress and signals from other cells; (3) two sensing units; (4) a communication module; (5) an oscillating gate of the stochastic switch which is regulated by the timer.

### The stochastic switch

At the heart of the decision of entrance into competence lies a positive feedback loop involving the master regulator ComK, which is kept at low levels by active degradation by degrader complex MecA (Figure 4C). Fluctuations can lead ComK to cross a threshold for self-activation and initiation of competence. This simple design implements a stochastic switch in which the odds of activation can be adjusted by controlling the basal levels of ComK. When the level of ComS is increased, a larger fraction of the degradation complex MecA is taken up by ComS degradation, allowing ComK levels to increase. This is the mechanism by which the level of ComS regulates the basal levels of ComK and consequently the probability of transitions into competence. Since the level of ComS is determined by information received from the sensing and communication units regarding the population density and stress, the transition probability of the individual cell is controlled by these order parameters describing the state of the group (population). In addition to the above, the stochastic switch is kept closed (ComK is inhibited) by a decision gate which is regulated to open by the phosphorylation timer.

### The adaptable timer

Since sporulation is a last resort, the cell needs to be sure to exhaust all other possibilities before the commitment. The circuit responsible for timing the initiation of sporulation (Figure 4D) integrates several different stress signals into a phosphorelay that ends in the activation of sporulation master regulator Spo0A. While high levels of activated Spo0A^*^ will irreversibly commit the cell to sporulation, intermediate levels will play a role in the selection of most other alternative phenotypes. The phosphorelay effectively works as an internal timer for the cell, which measures exposure to stress and coordinates phenotypic changes under the right circumstances. The timer clock rate of the individual cell is regulated by the experience of stress and information received from other cells (regarding the group stress). From a physics perspective, the adaptable timer presents an interesting element that has a “proper time” determined by an interplay between the local field (the stress sensed by the individual cell) and the group order parameter (the population stress). In addition, as is discussed later, the level of Spo0A^*^ feeds back and regulates (via the transcription factor σ^H^) the stress sensing system. Therefore, the local field at a given time is regulated by the time integral of the local stress and the population stress at previous time.

Note that since the dynamics of ComS (directly) and Spo0A^*^ (via the decision gate) affect the transition probability, in principle, an external observer cannot predict the decision of the individual cell unless it has information about the history of the entire population.

### The oscillating gate

Sporulation and competence are distinct fates that are induced by similar signals. Once the cell is on its path toward sporulation and Spo0A^*^ starts to accumulate, a decision gate (Figure 4E) links the decision of the two processes and opens a “window of opportunity” for transitions into competence. A special architecture with an incoherent loop of three consecutive inhibitions generates oscillations, opening the gate for short periods of time in which inhibition of the stochastic switch is lifted, thus giving rise to an increase in the transition probability (probability pulse).

### The integrated decision system

Figure 4A illustrates how the above modules are integrated together with the stress sensing unit, the cell density sensing unit and the communication unit into a decision system that will choose the cell's fate.

## The stochastic switch

The stochastic switch that controls entrance into competence, illustrated in Figure 4C, is composed of the master regulator ComK, a degradation complex MecA and a peptide ComS. Self-activation of ComK through a positive feedback loop requires its levels to rise above a certain threshold, but ComK levels are kept low by active degradation by MecA. At this stage, fluctuations in the basal expression of ComK are not sufficient to reach the threshold for self-activation. ComS has its production linked to quorum sensing signals, and competes for degradation with ComK by binding to the same degradation complex. When production of ComS is increased, a larger fraction of the degradation complex is taken up by ComS degradation, allowing ComK levels to increase. ComS expression effectively controls the basal levels of ComK and therefore also controls the probability that fluctuations will lead to self-activation and the transition to competence.

### Modeling the ComK-MecA-ComS core element

The operation of the ComK-MecA-ComS circuit has been studied in detail by modeling as a dynamical system with two variables—the concentrations of ComK and ComS (concentration of the degradation complex is fairly constant). It has been proposed that the ComK-MecA-ComS circuit can act as a bi-stable system, an excitable system, or both, depending on parameters (Maamar and Dubnau, 2005; Süel et al., 2006, 2007; Schultz et al., 2007; Leisner et al., 2008). Either excitable or bi-stable, the module acts as a stochastic switch, the action of which can be described as activation over an effective energy barrier, whose height is regulated by the concentration of ComS.

Here we focus on the transitions into competence and hence in the effect of ComS on ComK. It should be mentioned, for completeness, that the exit from competence back to the vegetative state is regulated by the inhibition of ComS by ComK (note that this inhibitory link is not shown in Figure 4C). Taking this inhibition into account, the stand-alone dynamics of the ComK-MecA-ComS circuit is modeled by,

(12) $$\begin{array}{l} \text{dS/dt = }{\text{g}}_{\text{S0}}\text{ + }{\text{g}}_{\text{SK}}{\text{H}}_{\text{SK}}^{-}(\text{K},\;{\text{n}}_{\text{SK}},\;{\text{K}}_{\text{0S}})\\ \;-\;\Lambda_{\text{S}}\text{S/}[\text{1 + }(\text{S/}\Gamma_{\text{S}})\text{ + }(\text{K/}\Gamma_{\text{K}})] \end{array}$$

(13) $$\begin{array}{l} {\text{dK/dt = g}}_{\text{K0}}\text{ + }{\text{g}}_{\text{KK}}{\text{H}}_{\text{KK}}^{+}(\text{K},\;{\text{n}}_{\text{KK}},\;{\text{K}}_{\text{0K}})\\ \;-\;\Lambda_{\text{K}}\text{K/}[\text{1 + }(\text{S/}\Gamma_{\text{S}})\text{ + }(\text{K/}\Gamma_{\text{K}})] \end{array}$$

**Figure 5:**
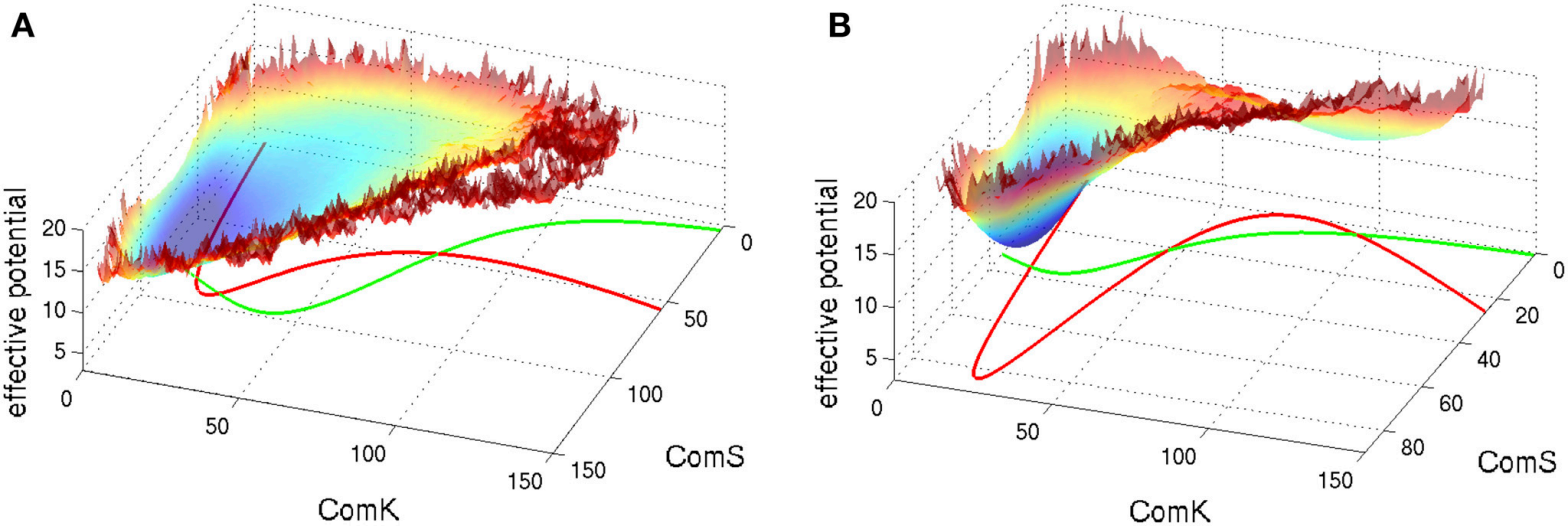
**Typical phase space for the standalone ComK-MecA-ComS model**. The nullclines for dK/dt = 0 are marked green lines and the ones for *dS/dt* = 0 are marked red lines. **(A)** For set of parameters which correspond to one stable fixed vegetative point and one unstable competent fixed point. **(B)** For set of parameters with bistability—coexistence of two metastable fixed points. The superimposed effective potential was evaluated (Schultz et al., 2007) by solving the stochastic equations (including the probability of binding and unbinding) to evaluate the probability density, P(K,S), at the ComK-ComS 2-dimentional phase space. The effective potential is defined as −log(P). Note that for **(A)** the effective potential shows a predominantly flat region corresponding to the excursions into competence and for **(B)** it shows two wells as the system can sporadically switch between the two metastable states (Adopted from Schultz et al., 2007).

S and K in these equations represent the number of peptides ComS and proteins ComK respectively. We note that these equations are written following the notation used here and they agree with the equations presented in Schultz et al. (2007) in the limit that the basal production rates g_S0_ and g_K0_ are small. Typical examples of the corresponding phase space of these equations are shown in Figure 5 superimposed with an effective potential which represents the effect of noise as is explained in the figure captions.

### The stochastic switch as a particle in a potential well

Here we are interested in the case that the production of ComS is regulated by the sensing system. Hence, S (ComS) is treated as a control parameter in Equation (13), which models the switch operation in this case. In this one dimensional case, the time dynamics of K (ComK) can be viewed as the overdamped (high friction) dynamics of a particle moving under the action of external force F(K,S), given by,

(14) dK/dt = F(K, S)

(15) $$\begin{array}{l} \text{F}(\text{K},\text{ S}){\text{ = g}}_{\text{K0}}\text{ + }{\text{g}}_{\text{KK}}{\text{H}}_{\text{KK}}^{+}(\text{K},\;{\text{n}}_{\text{KK}},\;{\text{K}}_{\text{0K}})\\ \;-\Lambda_{\text{S}}\text{S/}[\text{1 + }(\text{S/}\Gamma_{\text{S}})\text{ + }(\text{K/}\Gamma_{\text{S}})] \end{array}$$

Note that F(K,S) equals to the right hand side of Equation 13 for a fixed S. We note that when S is treated as a control parameter, the unstable competence fixed point becomes metastable. However, doing so has an effect on the transitions from the competence state and not on the transitions into the competence state which are the focus in this review. We use a set of parameters corresponding to bistable behavior in *K*, which could correspond to either the bistable or excitable model in a two-variable problem.

**Figure 6:**
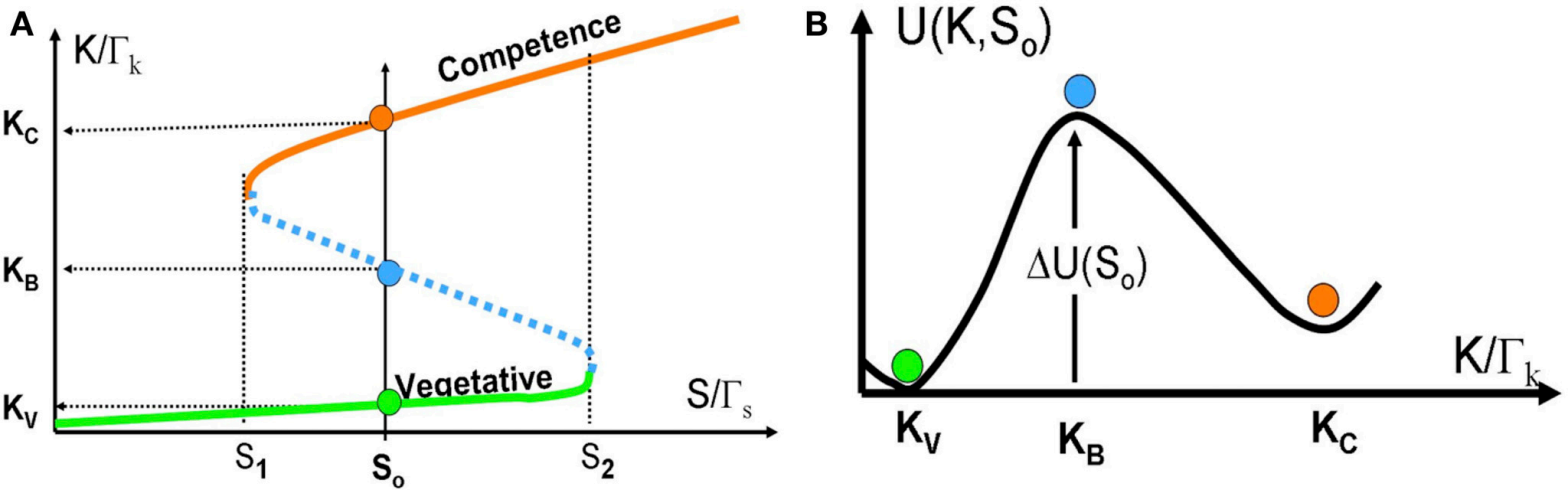
**The stochastic switch as a particle in a potential well. (A)** The bifurcation diagram as function of S acting as a control parameter described by Equations (14) and (15). The model parameters are selected such that the model exhibits bistability for S_1_ < S < S_2_. Details about the model parameters are given in Schultz et al. (2007, 2013). The red line shows the competence state that is associated with high ComK levels; the green line shows the vegetative state that is associated with low ComK levels. The dotted blue line shows the saddle points along the above two stable steady states. The green, red and blue circles in **(B)** illustrates the competence, vegetative and saddle point steady states respectively. **(B)** The effective potential [Equation (16) for a specific value *S* = *S*_0_ shown in **(A)**].

In Figure 6A we show the bifurcation diagram as function of S for the above 1-dimentional model. The bifurcation diagram reveals that below a certain concentration of ComS S_1_, vegetation (low concentrations of ComK) is the only stable state, and the system cannot enter competence. Above this threshold, the competence state, with high concentrations of ComK, coexists with the vegetative state. This bistability exists up to another concentration S_2_, above which the vegetative state does not exist. Usually, this threshold is well above the normal concentrations of ComS in bacteria.

For values of ComS such that S_1_ < S < S_2_, noise can induce spontaneous transition from the low K state to the high K state (from the vegetative to the competent state). From physics perspective, the transition can be described as transitions of an overdamped particle over a potential barrier (the high dissipation limit of Kramers theory) as is illustrated in Figure 6B. The potential and the potential barrier for a given value S_o_, are defined as follows:

(16) $$\text{dU}(\text{K},\;{\text{S}}_{\text{o}})\text{/dK }\equiv -\text{F}(\text{K},\;{\text{S}}_{\text{o}})$$

(17) $$\Delta \text{U}({\text{S}}_{\text{o}})\equiv -\int_{\text{Kv}-\text{>Kb}}\;{\text{[F(K',S}}_{\text{o}}\text{)]dK'}$$

Note that the “potential” U(K,S_o_) has units of [protein number]^2^/[time] as is explained in Schultz et al. (2013).

Exploiting the “particle in a well” picture and assuming a Gaussian white noise level ε, the transition probability per unit time τ, from the vegetation into the competence state, for a given value S = S_o_, is given by

(18) $$\tau =\Omega \exp[-\Delta U(S_{0})/\varepsilon ].$$

Ω is the attempt frequency according to Kramers theory in the high dissipation limit: **Ω** = **γω_V_/ω_B_** where **γ** is the effective friction and **ω_V_** and **ω_B_** are the second derivatives (frequencies) of the potential at K_V_ and K_B_ respectively. We emphasize that the effective noise is associated with binding and unbinding of ComK to its own promoter and the binding and unbinding of ComK and ComS to the complex MecA. Therefore, a more accurate approximation should involve extension of Kramers theory for the case of state dependent shot like noise. In either case the transition probability strongly depends on the potential barrier ΔU(S = S_o_). Since the potential barrier decays rapidly to zero from S = S_1_ to S = S_2_, the transition probability is very sensitive to the value of ComS as a control parameter. For systems with multiple components, similar formulism remains applicable, where the corresponding landscape can be defined as U = −log(P) (see Lu et al., 2014b, for a generic landscape construction method based on the WKB approximation).

### Collective control of the individual transition probability

Cell density and population stress control the level of ComS, and hence the transition probability of each individual cell. More specifically, the quorum sensing pheromone ComX, whose level is proportional to the population density, activates the production of ComS via the quorum sensing ComP-ComA two-component circuit (Comella and Grossman, 2005). The level of ComS is further controlled by the population stress through control of the ComP-ComA circuit by input received from the communication unit.

The collective control of competence transitions of individual cells by cell-cell signaling guarantees that the competence phenotype will only be selected at higher colony densities and high stress, when free DNA is more abundant in the environment. The regulation of competence initiation is therefore inserted in a context of high social complexity in the *B. subtilis* lifecycle, where accumulation of stress signals dictates cell decisions. In other words, the basal expression of ComK that determines the odds of competence transitions is ultimately regulated by the two order parameters that describe the state of the population. The values of these order parameters are sensed by all cells in a neighborhood, in order to collectively control the individual transition probabilities.

### Gating of the stochastic switch

While collective order parameters control the transition probability of the stochastic switch of an individual cell, a special decision gate composed by AbrB and Rok prevents competence transitions. The gate opens and allows transitions only after the cell has been exposed to sufficiently high stress, for sufficiently long time (as measured by the adaptable timer described in the next section). The special oscillating dynamics of the decision gate is presented in section The Decision Gate. The gate prevents the transition into competence by transcription inhibition of ComK by both of its genes—AbrB and Rok—independently (Figure 4E). When these effects are incorporated, Equation (15) is replaced by the following dynamical equation:

(19) $$\begin{array}{l} \text{dK/dt}=\left[{\text{g}}_{\text{K0}}\text{ + }{\text{g}}_{\text{KK}}{\text{H}}_{\text{KK}}^{+}(\text{K},\;{\text{n}}_{\text{KK}},\;{\text{K}}_{\text{0K}})\right]\Theta (\text{B},\text{R})\\ \;-\Lambda_{\text{S}}\text{S/}[\text{1 + }(\text{S/}\Gamma_{\text{S}})\text{ + }(\text{K/}\Gamma_{\text{S}})] \end{array}$$

(20) $$\Theta (\text{B},\text{R})\text{ = }{\text{H}}_{\text{KB}}^{-}(\text{B},\;{\text{n}}_{\text{KB}},\;{\text{B}}_{\text{0}}){\text{H}}_{\text{KR}}^{-}(\text{R},\;{\text{n}}_{\text{KR}},\;{\text{R}}_{\text{0}})$$

We note that the “ComK inhibition” I defined in Schultz et al. (2013) is equal to [1 − Θ(B, R)] (the rational in that article was that *I* = 0 when there is no inhibition). The above equations combined with the calculations of the transition probability are used in section The Decision Gate to evaluate the transition probability of the gated stochastic switch.

## The adaptable timer

### Biochemical background: sensing and information transfer by phosphorylation

Kinases are special enzymes that are able to transfer a phosphate group in a process called phosphorylation. Kinases can recognize and phosphorylate other kinases, in a cascade, until the phosphate group reaches the final target, usually activating or deactivating an effector protein by conformational changes (Bijlsma and Groisman, 2003; Veening et al., 2005). Phosphorylation cascades are a common strategy used by cells to integrate many levels of control into a process, forming networks where specialized enzymes can introduce, transfer, or remove phosphate groups from the cascade. These regulatory networks operate at much faster timescales than those involving the expression of genes. Kinases can use different amino acids to bind to the phosphate group, which means they can have different affinities. While some kinases divide their time between the phosphorylated and unphosphorylated state, other kinases transfer the phosphate immediately upon phosphorylation. The concentrations of the former are of great importance to the flow of phosphate through the system, while the concentrations of the latter are not important as long as they are abundant enough not to be rate-limiting. Some kinases can auto-phophorylate in response to signals and introduce phosphates into the cascade, while enzymes called phosphatases specialize in removing phosphate from their substrates. Phosphatases are often connected to communication modules and exert control over the flow of phosphates through the system.

A typical mechanism of control in response to a signal is the two-component system. It is composed of a histidine kinase and a response regulator. Histidine kinases have lower affinity for phosphate, transferring it immediately upon phosphorylation. They often auto-phosphorylate in response to signals. Response regulators are activated upon phosphorylation and carry out the effect in response to the signal. They are usually kinases or transcription factors. A typical operation of a two-component system starts with a histidine kinase *h*, undergoing auto-phosphorylation in response to a signal *s*. A response regulator *r* is quickly phosphorylated by *h* and becomes active.

### Time measurement by gene circuit

Several aspects need to be taken into consideration by the cell when choosing the correct time to sporulate. As soon as conditions worsen and the cell enters the stationary phase the clock starts ticking, but in an ever-changing environment where several noisy stress signals have to be taken into consideration, the cell needs to filter out the environmental noise. Therefore, the cell fate decision follows integrating several stress signals over sufficient time. Five different kinases respond to different stress signals. They integrate the information and transfer it to the timer via a cascade of phosphate propagation, which ends in phosphorylation of Spo0A.

### Biological proper time—collective normalization of the clock rate

Since Spo0A^*^ acts as an activator of Spo0A, the information transfer leads to a rapid progression of the effective “proper time” which is represented by the level of Spo0A^*^ (Shafikhani and Leighton, 2004; Schultz et al., 2009). From a physics perspective it means that the proper time is the time normalized by the experienced stress level. More specifically, the clock rate (rate of accumulation of Spo0A^*^), is adjusted according to the severity of the stress, as in an hourglass with an adjustable neck. It can even be set backwards in case of alleviation of the conditions. The phosphorelay is composed of two two-element circuits acting in series: a sensing two-component system (KinA-E – Spo0F) that measures cell stress, and a regulatory two-component circuit (Spo0B – Spo0A) that determines the entry into sporulation. KinA-E are five histidine kinases which autophosphorylate in response to different stress signals. This phosphate is quickly transferred to serine kinase Spo0F, which is subject to dephosphorylation by the communication unit according to information received from other cells. Histidine kinase Spo0B quickly shuttles phosphate between Spo0F and Spo0A, the final destination. As phosphate is transferred down the phosphorelay, it starts the accumulation of Spo0A^*^, which induces the production of a sporulation specific sigma factor σ^H^. σ^H^ activates the transcription of Spo0F, allowing higher information flow (larger flow of phosphate) through the system. Concentrations of the histidine kinases are not important, since they do not keep the phosphate very long, and are in abundance.

## The decision gate

The decision gate allows transition into competence only within a “window of opportunity” between two values of Spo0A^*^. The part of the decision gate constituted by Spo0A-AbrB-Spo0E oscillates within that window of opportunity. Spo0A^*^ is dephosphorylated by Spo0E, which is transcriptionally inhibited by AbrB, which in turn is transcriptionally inhibited by Spo0A^*^. These three genes form a special repressilator-like architecture (Elowitz and Leibler, 2000). The classical repressilator is a well-studied gene circuit consisting of a 3-gene inhibition loop, i.e., gene A represses gene B that represses gene C, which in turn represses A (ABC for short). The circuit has been implemented experimentally in a cell, and showed oscillatory behavior (Elowitz and Leibler, 2000).

### Transcription driven repressilator

To better understand the functional role of the various features of the Spo0A-AbrB-Spo0E circuit, we first inspect the dynamics of a classical ABC that is transcription driven by an external signal S. The deterministic equations of such driven repressilator are given by:

(21) $$\begin{array}{l} \text{dA/dt}=[{\text{g}}_{\text{A}}+{\text{g}}_{\text{AA}}{\text{H}}_{\text{AA}}^{+}(\text{A},\;{\text{n}}_{\text{AA}},\;{\text{A}}_{\text{0A}})\\ \;+\;{\text{g}}_{\text{AS}}\text{S}]{\text{H}}_{\text{AC}}^{-}(\text{C},\;{\text{n}}_{\text{AC}},\;{\text{C}}_{\text{0A}})-{\text{k}}_{\text{A}}\text{A} \end{array}$$

(22) $$\text{dB/dt}={\text{g}}_{\text{B}}{\text{H}}_{\text{BA}}^{-}(\text{A},\;{\text{n}}_{\text{BA}},\;{\text{A}}_{\text{0B}})-{\text{k}}_{\text{B}}\text{B}$$

(23) $$\text{dC/dt}={\text{g}}_{\text{C}}{\text{H}}_{\text{CB}}^{-}(\text{B},\;{\text{n}}_{\text{CB}},\;{\text{B}}_{\text{0C}})-{\text{k}}_{\text{C}}\text{C}$$

The gene base production rates are g_X_ (X stands for A, B and C respectively) and the corresponding protein degradation rates are k_X_. Note that Equation (21) contains a term for A self-activation.

**Figure 7:**
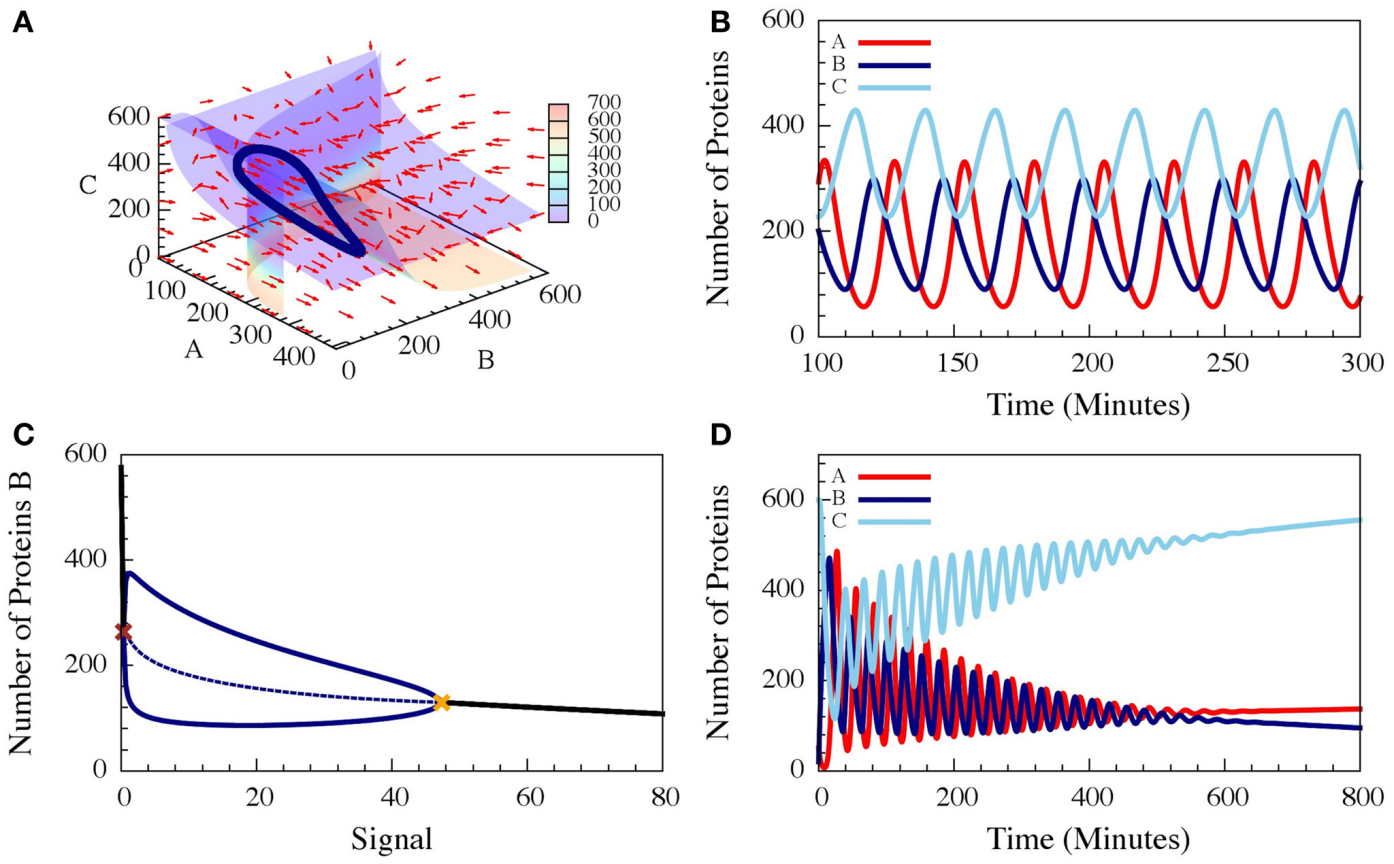
**The dynamics of a driven self-activating repressilator**. The results are for the model presented in Equations (21–23) when A is self-activating and is also linearly driven by an external input signal (Schultz et al., 2013). **(A)** The 3-diemnsional phase space for level of the input signal which corresponds to oscillating dynamics. The figure shows the nullsurfaces, the flow in the phase space and the limit cycle of the oscillating dynamics. **(B)** Time dependence of the number of proteins for the oscillating dynamics. **(C)** The bifurcation diagram as function of the input signal. The solid blue lines are the upper and lower bounds of the **(B)** levels during the stable oscillation, while the dashed blue line shows the mean **(B)** levels. The red cross and yellow cross show the left and right bifurcation points. **(D)** The dynamics when the input signal is increased in time from the oscillating to the non-oscillating regions of the bifurcation (Adopted from Schultz et al., 2013).

These three-coupled ordinary deterministic equations of the repressilator model, define a 3-dimensional phase space. When it comes to 3-dimensional phase space, the nullclines are replaced by nullsurfaces. For dA/dt = 0, dB/dt = 0 and dC/dt = 0. In Figure 7A we show the nullsurfaces, the flow in the phase space and the trajectory of the limit cycle for a specific value of the driven signal S for which oscillatory solution exist. The corresponding time dynamics of the number of proteins is show in Figure 7B. In Figure 7C we show the bifurcation diagram as function of the driving signal and in Figure 7D we shown the time dynamics when the signal is increased in time.

It has been shown (Schultz et al., 2013), that for specific choices of “realistic” circuit parameters, the oscillatory behavior start above a threshold signal level S_1_ and exist up to a second higher signal level S_2_. The “realistic parameters” used in the simulations were selected to fit typical protein concentrations and time scales of protein synthesis and degradation, and were scaled to fit the case of the *in vivo* engineered repressilator (Elowitz and Leibler, 2000) such that time is measured in minutes and the concentrations in number of proteins per cell (Schultz et al., 2013). A variant of the classical repressilator in which A is self-activated was also investigated since self-activation of Spo0A plays important role in the dynamics of the Spo0A-AbrB-Spo0E circuit.

### Phosphorylation driven repressilator

**Figure 8:**
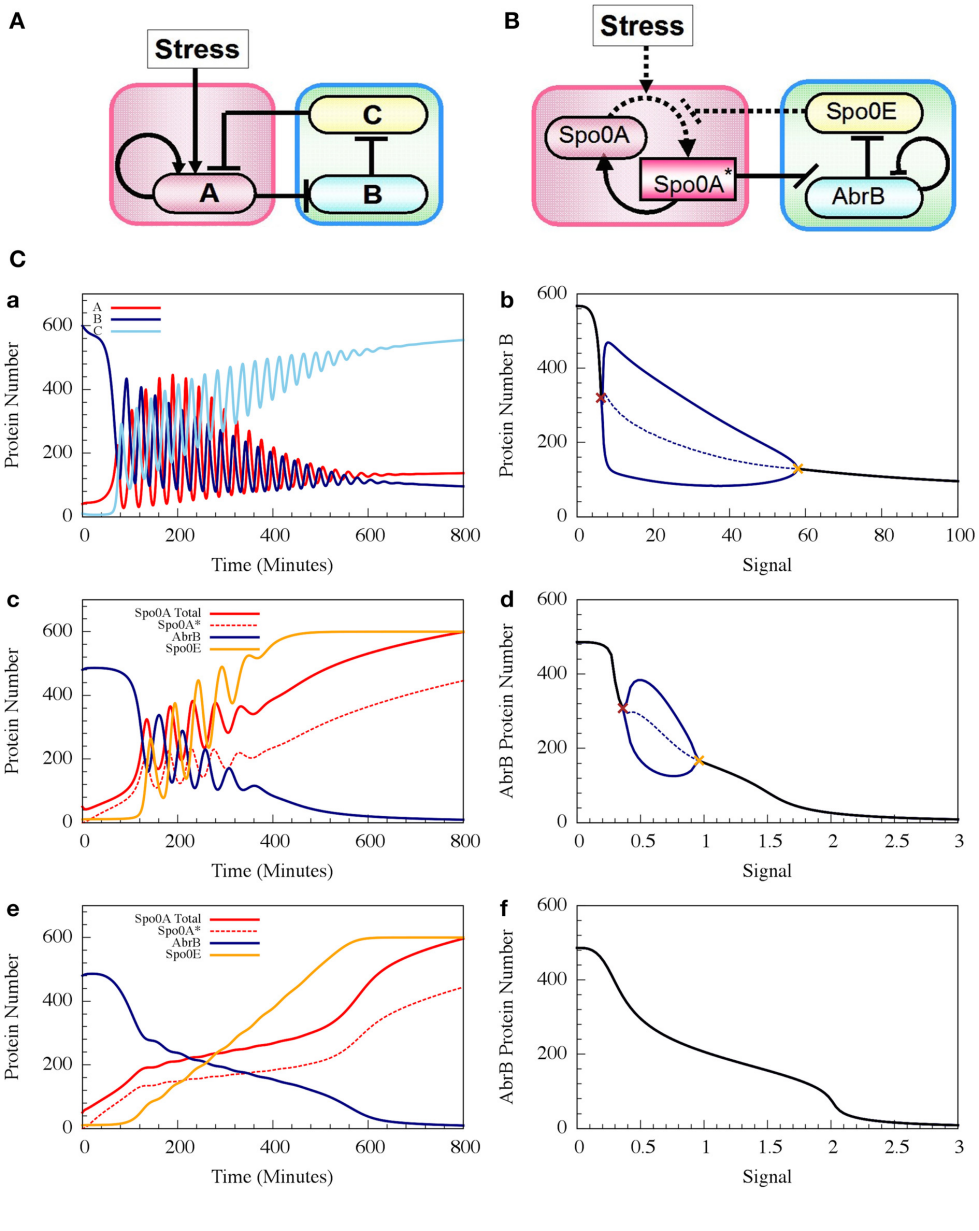
**The dynamics of transcription driven and phosphorylation driven repressilators. (A,B)** Show the two genetic circuits. **(C)** Shows their typical dynamical properties. **(a)** Time dynamics for the transcription driven repressilator circuit in **(A)**, where gene A is self-activation and we consider a Hill signal response (see Equation 10). The signal is linearly increased at rate 0.125 proteins per min. The plot shows levels of protein A (red solid line), B (navy solid line) and C (light blue solid line). **(b)** The bifurcation diagram associated to plot **(a)**. The plot shows the range of the protein B levels as the function of the constant signal level (X-axis). When the signal level is below or above the two bifurcation points as shown in brown and orange, the circuit is at a stable steady state (the protein level is shown in black). Between these bifurcation points, the circuit exhibits oscillation. The plot shows both the maximum and minimum levels of protein B (solid blue line) and the average levels (dotted blue line). **(c,d)** show similar plots but for the phosphorylation driven circuit in **(B)**, where circuit parameters yield oscillation (case I). **(e,f)** are similar to **(c,d)**, but for parameters that do not lead to oscillations (case II) (Adopted from Schultz et al., 2013).

The Spo0A-AbrB-Spo0E circuit can be regarded as a phosphorylation driven self-activating repressilator where A^*^ (Spo0A^*^), the phosphorylated Spo0A, inhibits B (AbrB) which inhibits E (Spo0E). Moreover, E dephosphorylates A^*^, while the input signal (I_S_ = S) phosphorylates A. A is activated by A^*^. The corresponding deterministic dynamics is described by the following coupled equations for A, A^*^, B and E:

(24) $$\begin{array}{l} \text{dA/dt}=[{\text{g}}_{\text{A}}+{\text{g}}_{\text{AA}}{\text{H}}_{\text{AA}}^{+}({\text{A}}^{*},\;{\text{n}}_{\text{AA}},\;{\text{A}}_{\text{0AA}}^{*})]\\ \;-\;{\text{ph}}_{\text{AS}}{\text{I}}_{\text{S}}\text{A}+{\text{dph}}_{{\text{A}}^{*}\text{E}}{\text{EA}}^{*}-{\text{k}}_{\text{A}}\text{A} \end{array}$$

(25) $${\text{dA}}^{*}\text{/dt}={\text{ph}}_{\text{AS}}{\text{I}}_{\text{S}}\text{A}-{\text{dph}}_{{\text{A}}^{*}\text{E}}{\text{EA}}^{*}-{\text{k}}_{{\text{A}}^{*}}{\text{A}}^{*}$$

(26) $$\begin{array}{l} \text{dB/dt}={\text{g}}_{\text{B}}{\text{H}}_{{\text{BA}}^{*}}^{-}({\text{A}}^{*},\;{\text{n}}_{{\text{BA}}^{*}},\;{\text{A}}_{{\text{OBA}}^{*}}^{*})\\ \;{\text{H}}_{\text{BB}}^{-}(\text{B},\;{\text{n}}_{\text{BB}},\;{\text{B}}_{\text{0BB}})-{\text{k}}_{\text{B}}\text{B} \end{array}$$

(27) $$\text{dE/dt}={\text{g}}_{\text{E}}{\text{H}}_{\text{EB}}^{-}(\text{B},\;{\text{n}}_{\text{EB}},\;{\text{B}}_{\text{0EB}})-{\text{k}}_{\text{E}}\text{E},$$

where ph_AS_ is the rate constant of the Spo0A phosphorylation by the input signal, and dph_A^*^E_ is the rate constant of Spo0A^*^ dephosphorylation by Spo0E. H^−^_BB_ (B, n_BB_, B_0BB_) is the inhibitory Hill function describing the self-inhibition of AbrB. In Figure 8 we show example of the oscillatory behavior of the Spo0A-AbrB-Spo0E circuit. This figure illustrates (as explained in Schultz et al., 2013) that the phosphorylation/dephosphorylation regulation, instead of transcription regulation, enables the confinement of the oscillations within a narrower time window. This permits better coordination between the decisions made by different cells. We also show in the figure that while the gate can exhibit oscillations for a wide range of parameters (as shown in Schultz et al., 2013), not all parameter sets generate oscillations.

### Turning oscillations into opportunities

**Figure 9:**
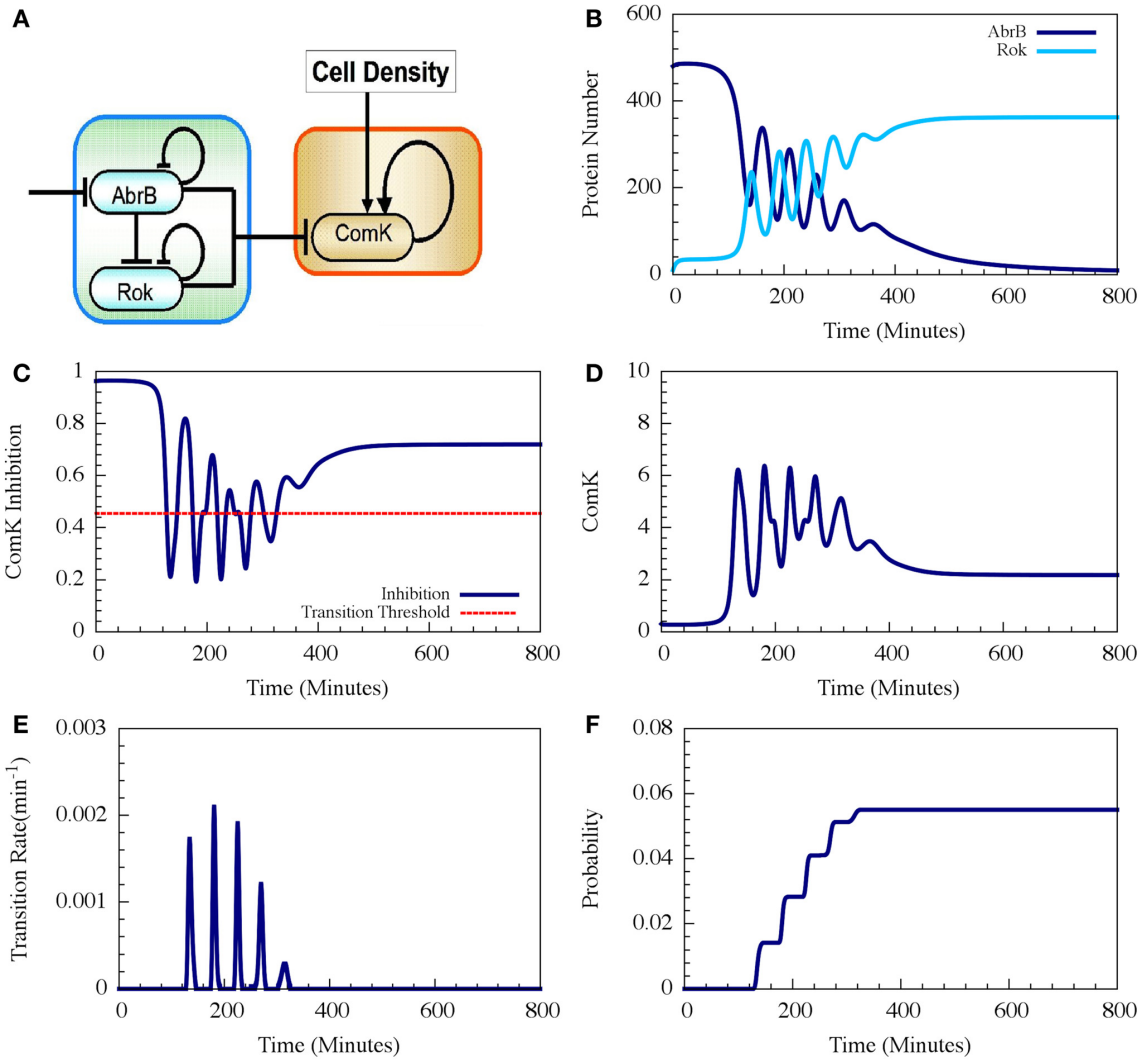
**Typical dynamics of the AbrB-Rok decision gate. (A)** The circuit diagram for the AbrB-Rok gating. **(B)** The reciprocal oscillatory dynamics of both the AbrB and Rok. **(C)** The time dynamics of the ComK Inhibition (*I* = [1 − Θ(B,R)]). The red dash line shows the threshold level of *I*, below which the system allows competence transition. **(D)** The time dynamics of ComK. **(E)** The corresponding transition probability per unit time showing the “opportunity spikes” produced at each oscillation. **(F)** The corresponding accumulated probability of competence transitions before commitment to sporulation (Adopted from Schultz et al., 2013).

AbrB and Rok are both self-inhibitory genes that also act as an inhibitor of ComK (Figure 9A). The dynamic equation describing the concentration of Rok models the inhibition of Rok by AbrB by an inhibitory Hill function H^−^_RB_(B, n_RB_, B_0_), which is multiplied by inhibitory Hill function H^−^_RR_(R, n_RR_, B_0_) representing the self-inhibition of Rok (Albano, 2005; Schultz et al., 2013). Thus, the deterministic dynamics of Rok is described by

(28) $$\text{dR/dt}={\text{g}}_{\text{R}}{\text{H}}_{\text{RB}}^{-}(\text{B},\;{\text{n}}_{\text{RB}},\;{\text{B}}_{0}){\text{H}}_{\text{RR}}^{-}(\text{R},\;{\text{n}}_{\text{RR}},\;{\text{R}}_{0})-{\text{k}}_{\text{R}}\text{R}.$$

Therefore, the oscillations in AbrB induce reciprocal oscillations in Rok but with some small phase shift (Figure 9B). At each oscillation there exists a short time interval during which both AbrB and RoK levels are sufficiently low that the mutual inhibition of ComK is lifted (the gate is opened) as is shown in Figure 9C. Each opening of the gate leads to a temporary increase in the basal concentration of ComK (Figure 9D), which in turn raises the probability of a transition into competence. In the presence of noise, the resulting increase in the level of ComK leads to an increase in the probability per unit time of transition into competence, as is shown in Figure 9E. The final outcome is that the decision gate opens a window of opportunity with several spikes of transition probability (one for each oscillation) and, hence, the accumulated transition probability increases in steps (Figure 9F).

In the example shown in Figure 9 the accumulated probability of competence transitions is about 0.06. Despite that the probability depends on the circuit parameters, we note that, with “realistic parameters,” the accumulated transition probability is typically between 0.01 and 0.1, which agrees well with experimental observations.

## Noise managements

Cell fate determinations are carried out with special capacity for different noise management by the different modules. For example, the ComK stochastic switch is driven by noise that is required for transitions into competence, where the ComK positive feedback loop is activated when fluctuations lead the ComK concentration to cross a certain threshold. On the other hand, the adaptable timer has to integrate stress signals over time and filter out environmental noise (Schultz et al., 2009, 2013). If the cell fails to do so, undesirable stress fluctuations would lead to a bad decision to sporulate at wrong times. The AbrB-Rok decision gate bridges these two modules that have opposite requirements of noise response. Therefore, efficient coordination between these modules calls for special noise management characteristic associated with distinct circuit architecture. In this section we show that the special architecture of the AbrB-Rok gate affords special noise management capabilities; it can efficiently filter out external noise and at the same time adds to the noise in the regulation of ComK (low concentration—high noise) (Ben-Jacob and Schultz, 2010). We also show that the circuit harnesses noise to be less sensitive to the circuit parameters thus utilizing noise to reduce the effect of cell-cell variability. Thus, we investigated the role of the AbrB-Rok gate in managing external and internal noise.

### Filtering out external noise

It has been shown that the cell decision-making between sporulation and competence is insensitive to external noise for the case where the parameters allow oscillations (such as the one presented in Figure 7). More specifically, we showed that noise on the input signal only slightly changes the accumulated transition probability into competence (Schultz et al., 2013). In other words, the circuit functions to integrate the stress signals while filtering out noise, guaranteeing a robust response. In Figure 9 we show that addition of external noise to a system with non-oscillating parameters (such as the one presented in Figure 7) leads to oscillations, yet the accumulated transition probability is only slightly affected.

### Management of internal noise

We used a stochastic approach to investigate the management of internal noise for the AbrB-Rok gate. Here, instead of using differential equations that depict the deterministic dynamics, we modeled the system by considering protein binding and unbinding, synthesis and degradation as stochastic events. The probabilities of having each stochastic event are set to match the rate constants in the deterministic equations, while the noise level is adjusted by choosing different levels of binding and unbinding rates, taken from Schultz et al. (2013). The system was then simulated with the Gillespie algorithm (Gillespie, 1977).

**Figure 10:**
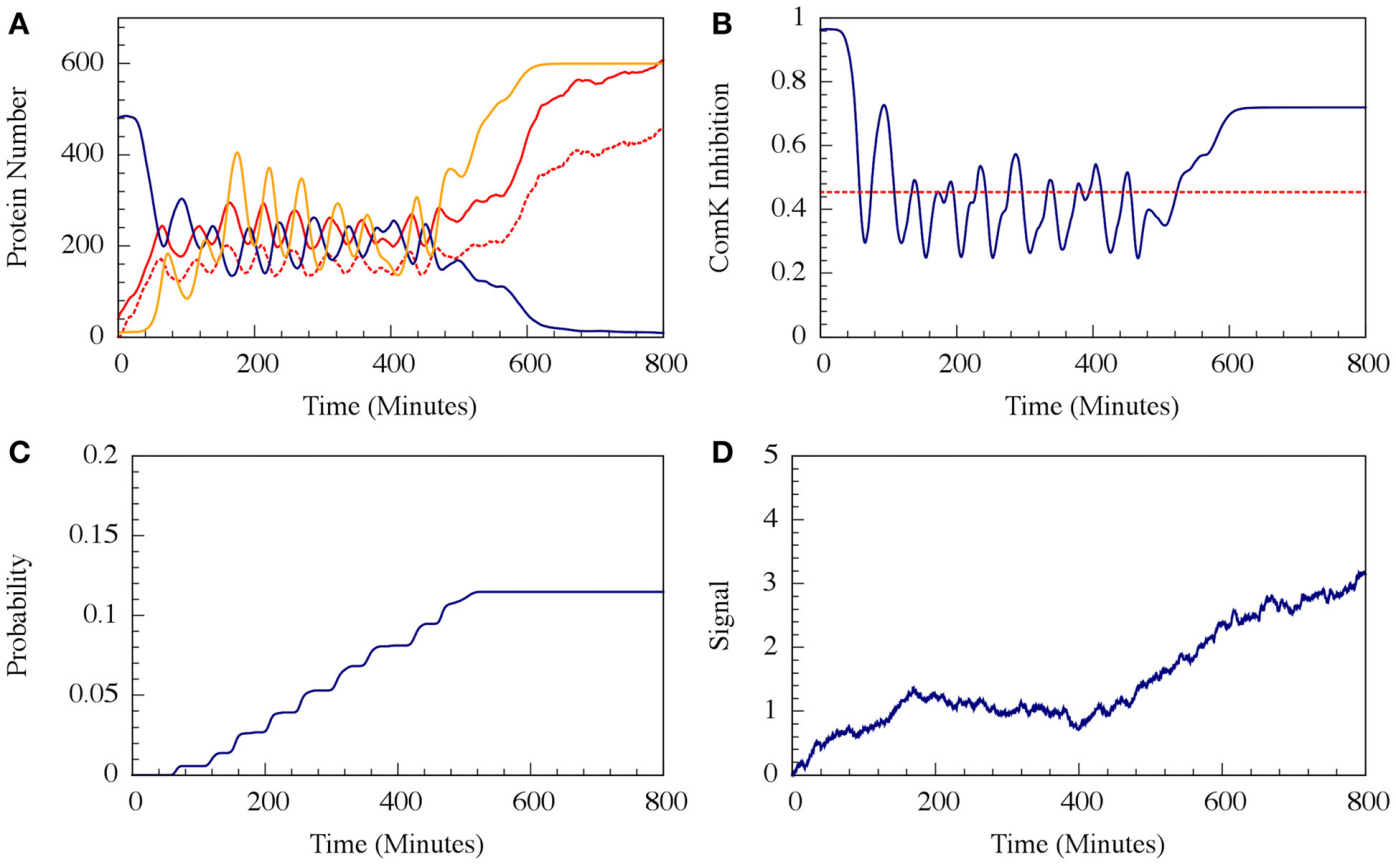
**The effect of external noise for the non-oscillating case shown in Figure 8**. **(A)** A typical dynamical behavior—dynamics of protein levels for all Spo0A (including both Spo0A and Spo0A^*^, solid red line), Spo0A^*^ (dash red line), AbrB (solid navy line) and Spo0E (solid yellow line). **(B)** The corresponding dynamics of the ComK inhibition *I* = [1 − Θ(B,R)] defined in Equation (20). **(C)** The accumulated transition probability into competence before commitment to sporulation. **(D)** Time dynamics of the input signal I. The I value is zero at time 0, and approaches to about 3 (in A.U.) at time 800 mins. (Adopted from Schultz et al., 2013).

**Figure 11:**
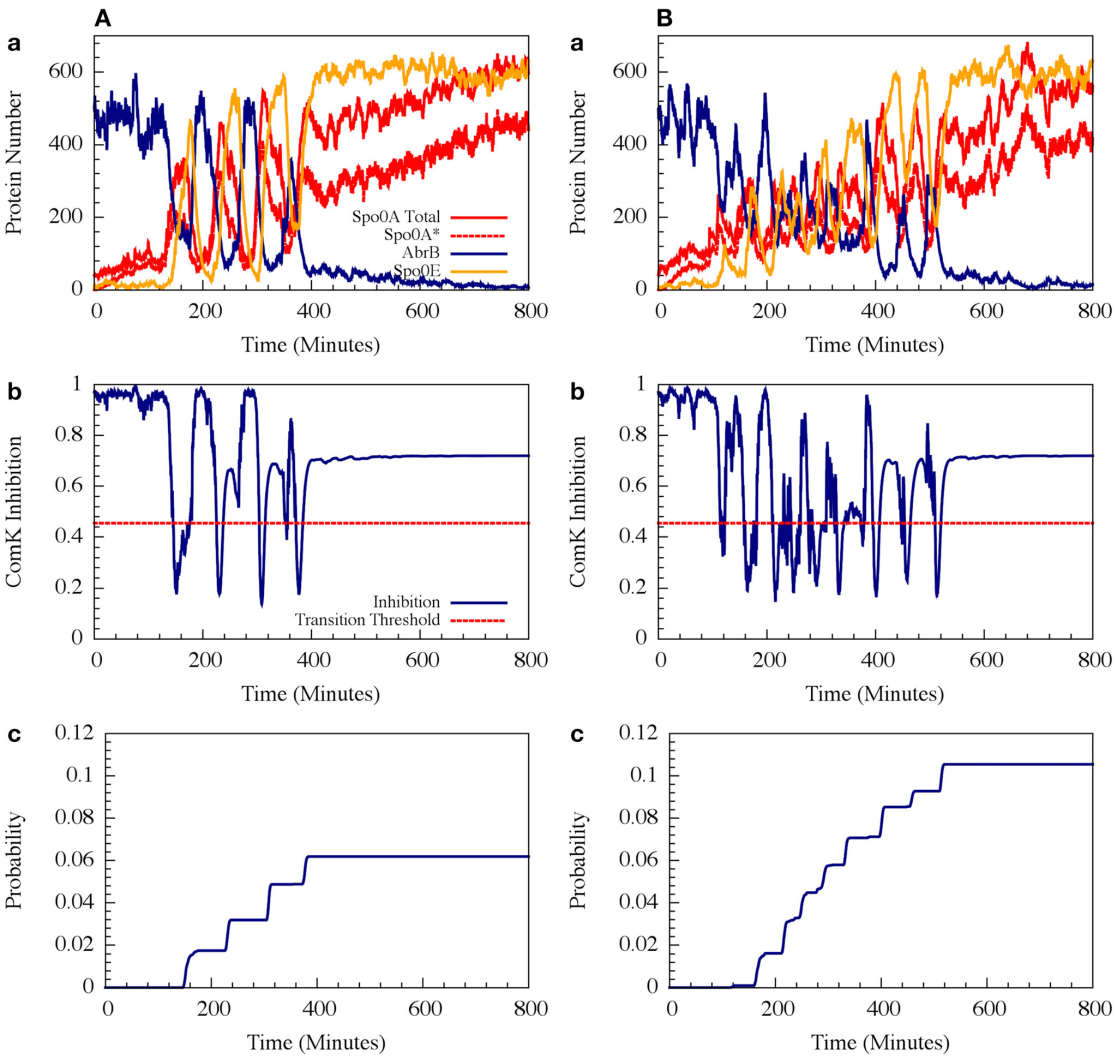
**The effects of internal noise on the circuit dynamics**. Panel **(A)** Is for the oscillating case; Panel **(B)** is for the non-oscillating **(b)** case. The simulations were performed by Gillespie algorithm. Panels **(a)** show the dynamics of the protein levels for each gene for specific realization where unbinding rate is 5 per min for both the oscillating and the non-oscillating cases shown in Figure 7. Panels **(b)** show the dynamics of the ComK Inhibition *I* = [1 − Θ(B,R)], defined in Equation (20). Panels **(c)** show the accumulated transition probabilities for competence (Adopted from Schultz et al., 2013).

**Figure 12:**
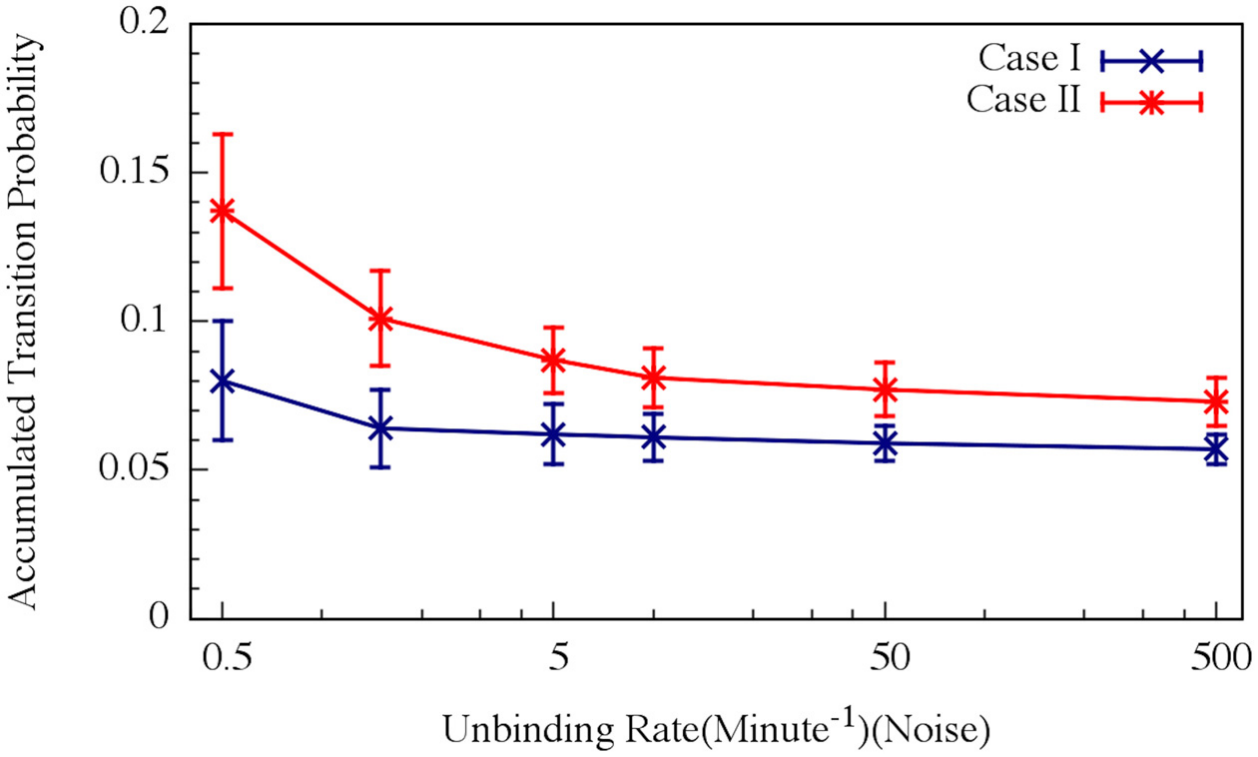
**Effects of the unbinding rates on the accumulated transition probability for the oscillating (Case I) and non-oscillating (Case II) cases**. See Schultz et al. (2013) for details. Case I: crosses and navy line (shown in Figure 8A); Case II: stars and red line (shown in Figure 8B) (Adopted from Schultz et al., 2013).

For circuit parameters which allow oscillations in the deterministic case (the oscillatory case), the internal noise makes the oscillations to be less ordered, yet accumulated transition probability is kept almost unchanged, as is illustrated in Figures 11, 12 (Schultz et al., 2013). For parameters that do not show oscillatory dynamics in the deterministic case, the internal noise has a greater effect. It can induce oscillations that look similar to those for the case where the deterministic dynamics is oscillatory, as is shown in Figure 10. Yet, the noise has only weak effect on the accumulate transition probability as is shown in Figure 11. So, the unique architecture of the AbrB-Rok gate allows the system to have oscillatory dynamics even when there is no oscillation in the deterministic limit. Moreover, the special noise management makes the dynamics more robust against variations in the circuit parameters.

The results in this section illustrate the special noise management capabilities of the decision gate. The special architecture of this element leads to oscillations that are less sensitive to circuit parameters and less sensitive to internal noise. For parameters whose deterministic dynamics is not oscillating, noise can generate oscillations, rendering the operation of the gate to be less sensitive to cell-cell variations in circuit parameters. Being driven by phosphorylation instead of transcription, the repressilator has a narrow and well-defined “window of opportunity” and is less sensitive to fluctuations in external stress.

## Toward population level—looking at the ensemble

### Global description of the communication unit

In order to synchronize neighboring cells, additional mechanisms exist where the cell sends and receive signals that are informative of their stress levels. The ones that are of particular interest for the decision between competence and sporulation are the quorum sensing (described earlier) and the Rap system. The Rap system expresses a series of phosphatases that act by slowing down the accumulation of stress and also exports matching signaling peptides that inactivate these same phosphatases (Core and Perego, 2003; Smits, 2007; Bischofs et al., 2009; Schultz et al., 2009). These signals are then averaged in the environment and re-imported to regulate the action of the phosphatases, guaranteeing robustness of the colony against cell-to-cell variations. There are over 11 different pairs of Rap proteins and their matching pheromones identified in *B. subtilis*, including many redundant ones. We will illustrate their mechanism by the showing the role of RapA. When a cell senses stress, it starts speeding in the path to sporulation by accumulating Spo0A^*^. RapA is induced by quorum sensing and slows down the accumulation of Spo0A^*^ by dephosphorylating Spo0F^*^. At the same time, the matching pheromone PhrA is exported to the environment. As the concentration of PhrA in the environment reaches convenient levels, all cells have their RapA proteins inactivated at the same time, guaranteeing a synchronized entrance into sporulation.

### Simplified modeling of cell-cell communication as exchange interactions

Despite the complicated nature of these pathways, one can simplify the modeling of cell-cell communication by introducing feedback terms to the input signal. The feedback term is modeled to be proportional to the master regulator of the process, with levels taken from all interacting cells and with a time delay to account for the processes in the signal transduction. Intuitively, the time delay for inter-cellular communication should be larger than that for the intra-cellular communication because of the diffusion of the pheromones.

**Figure 13:**
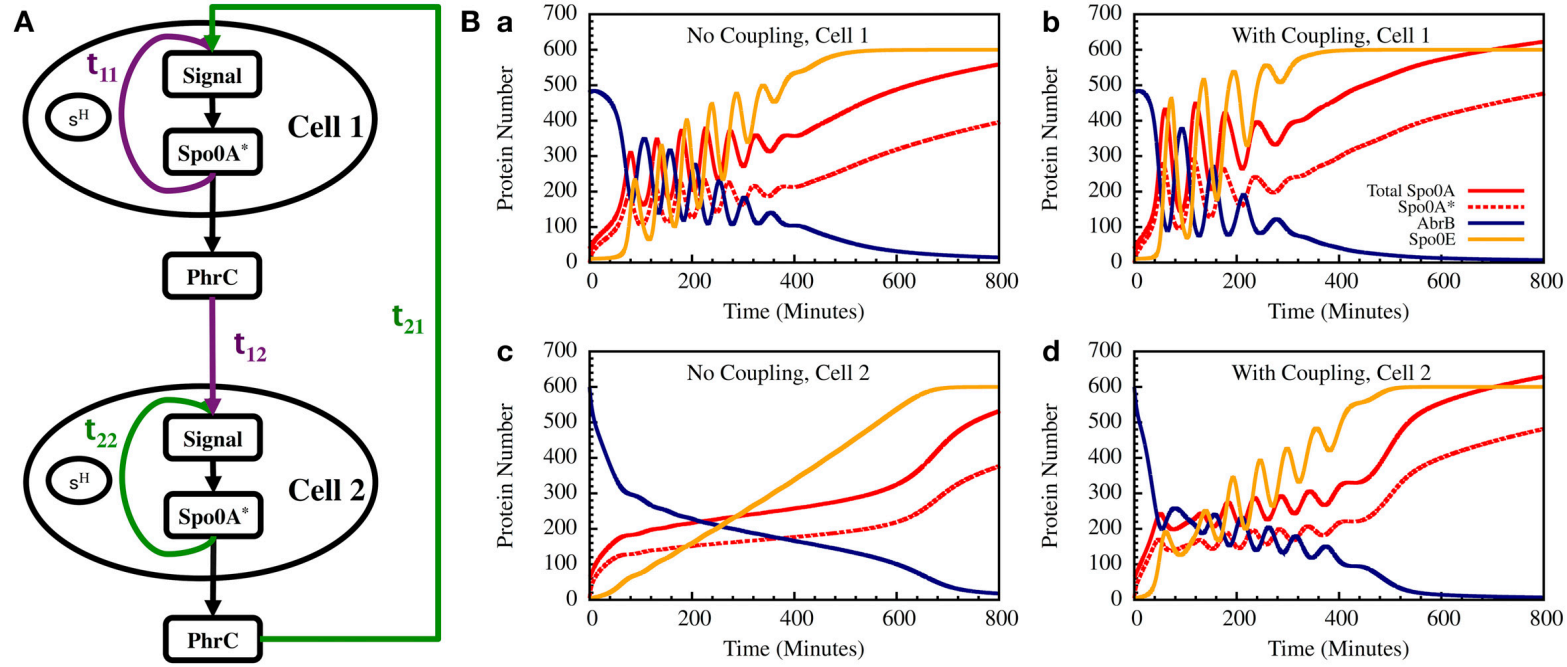
**The gate dynamics of two coupled cells. (A)** The schematic circuit illustrating the coupling between the two cells and the feedback from the timer to the sensing system. **(B)** Left—The standalone dynamics of an oscillating and a non-oscillating cell. Right—The dynamics of the two cells when they are coupled.

As a step toward modeling the effect of cell-cell communication we present here a simple example of two interacting cells as is illustrated in Figure 13A. The equations for the input signals for each of the cells, including input from the other cell and feedback from Spo0A^*^ (A^*^), are given by

(29) $${\text{S}}_{1}(\text{t})=\text{c}+\text{mt}+{\text{q}}_{11}{\text{A}}_{1}^{*}(\text{t}-\tau_{2})+{\text{q}}_{21}{\text{A}}_{2}^{*}(\text{t}-\tau_{1})$$

(30) $${\text{S}}_{2}(\text{t})=\text{c}+\text{mt}+{\text{q}}_{12}{\text{A}}_{2}^{*}(\text{t}-\tau_{2})+{\text{q}}_{22}{\text{A}}_{1}^{*}(\text{t}-\tau_{1}),$$

where c + mt is the linear signal initially presented in the single cell modeling of the Spo0A circuit, c = 0.25, m = 0.0028, τ_1_ = 10 min, τ_2_ = 5 min, and q_ij_ (i, j = 1, 2) are four coupling constants. Here, we assume q_11_ = q_22_ = q, q_12_ = q_21_ = 0.8q. The equations for the Spo0A circuit are the same as Equations (24)–(27).

In Figure 13B we show the results for a special case in which the uncoupled cell 1 is oscillating while cell 2 has non-oscillating stand-alone dynamics. We found that coupling between the two cells induce oscillations in both cells within similar “window of opportunity.” These results illustrate the special architecture of the decision gate coupled with the communication unit leading to coordinated decisions.

## Conclusions

Here we introduced the physics of bacterial decision-making using the intricate network used by *B. subtilis* for fate determination between sporulation and competence as our guiding example. The idea was to illustrate that taking physics approach enables us to simplify the complexity of the integrated decision network by identifying the basic functional modules and to study the dynamics of each module by further reduction into very simple gene circuits of only two or three components. From a physics perspective, one of the great challenges posed by collective decisions is to understand the special interplay between the individual cell and the population as a whole. More specifically, the challenge is to understand the noise management feature which leads to coordinated (or collectively controlled) stochastic decisions on the single cell level with predicted outcome on the population level. While each cell is freely to choose its own fate, the sporulation/competence ratio is collectively regulated to suit current conditions according to the requirement of the colony as a whole.

We showed that the decision-making during stochastic cell differentiations has a special capacity of noise managements at different circuit modules and different times in line with a desired function. For instance, it is undesirable to have noise in the adaptable timer (in the Spo0A phosphorylation pathway). Fluctuations in stress experienced by the individual cell must not lead to a decision to sporulation at inconvenient times since it could be harmful to the whole colony. Thus, the system has evolved to filter out transient activations and guarantee a robust response by integrating stress signals over time (Schultz et al., 2009). On the other hand, noise drives the ComK stochastic switch, where the ComK positive feedback loop is only activated when fluctuations in the ComK concentration lead it to cross a certain threshold.

An important yet less studied aspect of the sporulation/competence decision system is the AbrB-Rock decision gate, whose task is to regulate the opening/closing of the ComK stochastic switch based on the Spo0A timer. Efficient performance of coordination between two modules with opposite noise requirements calls for distinct circuit architecture with special dynamics and noise management characteristic.

Taking a physics approach, we searched for general principles and suggested that physics modeling helps to understand the operation-architecture principles of the decision gate: (1) “Inhibition of inhibition”—Spo0A^*^ inhibits the gate while the gate further inhibits ComK. (2) Circuit motif of phosphorylation driven repressilator. (3) “Window of opportunity” with oscillatory dynamics. “Inhibition of inhibition” has the advantage in that the gate is insensitive to the noise from Spo0A^*^ (high concentration—low noise) and meanwhile it adds to the noise in the ComK regulation (low concentration—high noise) (Ben-Jacob and Schultz, 2010). Since each oscillation increases the transition probability to competence of each cell by steps, we believe it has the advantage to improve coordination between cells as is discussed below.

As was mentioned earlier, the noise management of the gate makes its operation to be less sensitive to the circuit parameters (which describes cell-cell variations). Being phosphorylation driven (rather than transcription driven), the repressilator has a narrower and more well-defined “window of opportunity.” Besides, it is less sensitive to noise in the external stress.

Because neither sporulation nor competence is beneficial to a single cell, it is crucial that the decision to commitment to sporulation vs. escape into competence is made within a time frame that is coordinated with the other cells. Here, the phosphorylation driven repressilator motif enables narrow windows with the oscillating dynamics, which is important for cell-cell coordination. At each oscillation the cell secrets a pheromone signal when level of Spo0A^*^ (through the regulation of the Rap communication module) increases. Meanwhile, Spo0A^*^ has a positive feedback with time delay via its activation of Spo0F, and the cell receive signals from the other cells.

Looking ahead, the novel principles discovered in the context of sporulation vs. competence decision-making are likely to be relevant to some other cases of collective decisions of stressed bacteria, such as fratricide, cannibalism, and spore germination if conditions are improved. The adoption of different phenotypes by genetically similar members of the colony has interesting aspects relating to game theory. The adoption of a certain phenotype brings advantages and comes at a cost to the cell, and these advantages and costs depend not only on the environment, but also on the decisions of other cells in the colony. Decisions about the cell's fate are made in negotiations with neighboring cells, corresponding to a game where the players exchange information with each other. Strategies are not necessarily optimized by the cells personal interests, but also by the common good of the colony. Unlike most games, time is an important aspect of the formulation of bacterial decision-making. In the case of sporulation, for instance, the cell needs to choose the right time to initiate the highly energy consuming process. If a cell sporulates too early, conditions might improve and leave the cell abandoned by their peers. If a cell waits in a low energy consumption state, there might be less competition in case conditions get better. If a cell waits too long, there might not be enough resources left to complete the sporulation process. It is also worth mentioning that physics-based approach can be applied to understand the fat determination of some other biological systems, such as meiosis differentiation in yeast (Nachman et al., 2007), Epithelial-to-mensenchymal transitions in metastatic cancer (Lu et al., 2013a,b, 2014a; Tian et al., 2013; Zhang et al., 2014) and phenotypic variability in mammalian progenitor cells (Chang et al., 2008; Wang et al., 2011).

### Conflict of interest statement

The authors declare that the research was conducted in the absence of any commercial or financial relationships that could be construed as a potential conflict of interest.

## References

[B1] Acar M. Mettetal J. T. van Oudenaarden A. (2008). Stochastic switching as a survival strategy in fluctuating environments. Nat. Genet. 40, 471–475. 10.1038/ng.110 18362885

[B2] Aguilar C. Vlamakis H. Losick R. Kolter R. (2007). Thinking about *Bacillus subtilis* as a multicellular organism. Curr. Opin. Microbiol. 10, 638–643. 10.1016/j.mib.2007.09.006 17977783 PMC2174258

[B3] Albano M. (2005). The Rok protein of Bacillus subtilis represses genes for cell surface and extracellular functions. J. Bacteriol. 187, 2010–2019. 10.1128/JB.187.6.2010-2019.2005 15743949 PMC1064057

[B4] Be'er A. Florin E.-L. Fisher C. R. Swinney H. L. Payne S. M. (2011). Surviving bacterial sibling rivalry: inducible and reversible phenotypic switching in paenibacillus dendritiformis. MBio 2, e00069–11. 10.1128/mBio.00069-11 21628502 PMC3104493

[B5] Be'er A. Zhang H. P. Florin E.-L. Payne S. M. Ben-Jacob E. Swinney H. L. (2009). Deadly competition between sibling bacterial colonies. Proc. Natl. Acad. Sci. U.S.A. 106, 428–433. 10.1073/pnas.0811816106 19129489 PMC2626719

[B6] Ben-Jacob E. Coffey D. S. Levine H. (2012). Bacterial survival strategies suggest rethinking cancer cooperativity. Trends Microbiol. 20, 403–410. 10.1016/j.tim.2012.06.001 22750098

[B7] Ben-Jacob E. Schultz D. (2010). Bacteria determine fate by playing dice with controlled odds. Proc. Natl. Acad. Sci. U.S.A. 107, 13197–13198. 10.1073/pnas.1008254107 20660309 PMC2922178

[B8] Bijlsma J. J. E. Groisman E. A. (2003). Making informed decisions: regulatory interactions between two-component systems. Trends Microbiol. 11, 359–366. 10.1016/S0966-842X(03)00176-8 12915093

[B9] Bischofs I. B. Hug J. A. Liu A. W. Wolf D. M. Arkin A. P. (2009). Complexity in bacterial cell–cell communication: quorum signal integration and subpopulation signaling in the Bacillussubtilis phosphorelay. Proc. Natl. Acad. Sci. U.S.A. 106, 6459–6464. 10.1073/pnas.0810878106 19380751 PMC2672556

[B10] Cagatay T. Turcotte M. Elowitz M. B. Garcia-Ojalvo J. Süel G. M. (2009). Architecture-dependent noise discriminates functionally analogous differentiation circuits. Cell 139, 512–522. 10.1016/j.cell.2009.07.046 19853288

[B11] Chang H. H. Hemberg M. Barahona M. Ingber D. E. Huang S. (2008). Transcriptome-wide noise controls lineage choice in mammalian progenitor cells. Nature 453, 544–547. 10.1038/nature06965 18497826 PMC5546414

[B12] Comella N. Grossman A. D. (2005). Conservation of genes and processes controlled by the quorum response in bacteria: characterization of genes controlled by the quorum sensing transcription factor ComA in *Bacillus subtilis*. Mol. Microbiol. 57, 1159–1174. 10.1111/j.1365-2958.2005.04749.x 16091051

[B13] Core L. Perego M. (2003). TPR-mediated interaction of RapC with ComA inhibits response regulator-DNA binding for competence development in *Bacillus subtilis*. Mol. Microbiol. 49, 1509–1522. 10.1046/j.1365-2958.2003.03659.x 12950917

[B14] Elowitz M. B. Leibler S. (2000). A synthetic oscillatory network of transcriptional regulators. Nature 403, 335–338. 10.1038/35002125 10659856

[B15] Gillespie D. T. (1977). Exact stochastic simulation of coupled chemical reactions. J. Phys. Chem. 81, 2340–2361. 10.1021/j100540a008

[B16] Kaern M. Elston T. C. Blake W. J. Collins J. J. (2005). Stochasticity in gene expression: from theories to phenotypes. Nat. Rev. Genet. 6, 451–464. 10.1038/nrg1615 15883588

[B17] Kittisopikul M. Süel G. M. (2010). Biological role of noise encoded in a genetic network motif. Proc. Natl. Acad. Sci. U.S.A. 107, 13300–13305. 10.1073/pnas.1003975107 20616054 PMC2922135

[B18] Kollmann M. Løvdok L. Bartholomé K. Timmer J. Sourjik V. (2005). Design principles of a bacterial signaling network. Nature 438, 504–507. 10.1038/nature04228 16306993

[B19] Kuchina A. (2011). Temporal competition between differentiation programs determines cell fate choice. Mol. Syst. Biol. 7, 557. 10.1038/msb.2011.88 22146301 PMC3737729

[B20] Leisner M. Stingl K. Frey E. Maier B. (2008). Stochastic switching to competence. Curr. Opin. Microbiol. 11, 553–559. 10.1016/j.mib.2008.09.020 18955155

[B21] Lopez D. Kolter R. (2010). Extracellular signals that define distinct and coexisting cell fates in *Bacillus subtilis*. FEMS Microbiol. Rev. 34, 134–149. 10.1111/j.1574-6976.2009.00199.x 20030732

[B22] Losick R. Desplan C. (2008). Stochasticity and cell fate. Science 320, 65–68. 10.1126/science.1147888 18388284 PMC2605794

[B23] Lu M. Jolly M. K. Gomoto R. Huang B. Onuchic J. Ben-Jacob E. (2013a). Tristability in cancer-associated microRNA-TF chimera toggle switch. J. Phys. Chem. B 117, 13164–13174. 10.1021/jp403156m 23679052

[B24] Lu M. Jolly M. K. Levine H. Onuchic J. N. Ben-Jacob E. (2013b). MicroRNA-based regulation of epithelial–hybrid–mesenchymal fate determination. Proc. Natl. Acad. Sci. U.S.A. 110, 18144–18149. 10.1073/pnas.1318192110 24154725 PMC3831488

[B25] Lu M. Jolly M. K. Onuchic J. Ben-Jacob E. (2014a). Toward decoding the principles of cancer metastasis circuits. Cancer Res. 74, 4574–4587. 10.1158/0008-5472.CAN-13-3367 25183783

[B26] Lu M. Onuchic J. Ben-Jacob E. (2014b). Construction of an effective landscape for multistate genetic switches. Phys. Rev. Lett. 113, 078102. 10.1103/PhysRevLett.113.078102 25170733

[B27] Maamar H. Dubnau D. (2005). Bistability in the *Bacillus subtilis* K-state (competence) system requires a positive feedback loop. Mol. Microbiol. 56, 615–624. 10.1111/j.1365-2958.2005.04592.x 15819619 PMC3831615

[B28] Maamar H. Raj A. Dubnau D. (2007). Noise in gene expression determines cell fate in Bacillus subtilis. Science 317, 526–529. 10.1126/science.1140818 17569828 PMC3828679

[B29] Nachman I. Regev A. Ramanathan S. (2007). Dissecting timing variability in yeast meiosis. Cell 131, 544–556. 10.1016/j.cell.2007.09.044 17981121

[B30] Novák B. Tyson J. J. (2008). Design principles of biochemical oscillators. Nat. Rev. Mol. Cell Biol. 9, 981–991. 10.1038/nrm2530 18971947 PMC2796343

[B31] Raj A. van Oudenaarden A. (2008). Nature, nurture, or chance: stochastic gene expression and its consequences. Cell 135, 216–226. 10.1016/j.cell.2008.09.050 18957198 PMC3118044

[B32] Ray J. C. Tabor J. J. Igoshin O. A. (2011). Non-transcriptional regulatory processes shape transcriptional network dynamics. Nat. Rev. Microbiol. 9, 817–828. 10.1038/nrmicro2667 21986901 PMC3755963

[B33] Schultz D. Jacob E. B. Onuchic J. N. Wolynes P. G. (2007). Molecular level stochastic model for competence cycles in *Bacillus subtilis*. Proc. Natl. Acad. Sci. U.S.A. 104, 17582–17587. 10.1073/pnas.0707965104 17962411 PMC2077070

[B34] Schultz D. Lu M. Stavropoulos T. Onuchic J. Ben-Jacob E. (2013). Turning oscillations into opportunities: lessons from a bacterial decision gate. Sci. Rep. 3, 1668. 10.1038/srep01668 23591544 PMC3627974

[B35] Schultz D. Onuchic J. N. Ben Jacob E. (2012). Turning death into creative force during biofilm engineering. Proc. Natl. Acad. Sci. U.S.A. 109, 18633–18634. 10.1073/pnas.1215227109 23118336 PMC3503156

[B36] Schultz D. Wolynes P. G. Ben Jacob E. Onuchic J. N. (2009). Deciding fate in adverse times: sporulation and competence in *Bacillus subtilis*. Proc. Natl. Acad. Sci. U.S.A. 106, 21027–21034. 10.1073/pnas.0912185106 19995980 PMC2795487

[B37] Shafikhani S. H. Leighton T. (2004). AbrB and Spo0E control the proper timing of sporulation in *Bacillus subtilis*. Curr. Microbiol. 48, 262–269. 10.1007/s00284-003-4186-2 15057450

[B38] Sirota-Madi A. Olender T. Helman Y. Ingham C. Brainis I. Roth D.. (2010). Genome sequence of the pattern forming *Paenibacillus vortex* bacterium reveals potential for thriving in complex environments. BMC Genomics 11:710. 10.1186/1471-2164-11-710 21167037 PMC3012674

[B39] Smits W. K. (2007). Temporal separation of distinct differentiation pathways by a dual specificity Rap-Phr system in *Bacillus subtilis*. Mol. Microbiol. 65, 103–120. 10.1111/j.1365-2958.2007.05776.x 17581123

[B40] Süel G. M. Garcia-Ojalvo J. Liberman L. M. Elowitz M. B. (2006). An excitable gene regulatory circuit induces transient cellular differentiation. Nature 440, 545–550. 10.1038/nature04588 16554821

[B41] Süel G. M. Kulkarni R. P. Dworkin J. Garcia-Ojalvo J. Elowitz M. B. (2007). Tunability and noise dependence in differentiation dynamics. Science 315, 1716–1719. 10.1126/science.1137455 17379809

[B42] Tian X.-J. Zhang H. Xing J. (2013). Coupled reversible and irreversible bistable switches underlying TGFβ-induced epithelial to mesenchymal transition. Biophys. J. 105, 1079–1089. 10.1016/j.bpj.2013.07.011 23972859 PMC3752104

[B43] Veening J. Hamoen L. W. Kuipers O. P. (2005). Phosphatases modulate the bistable sporulation gene expression pattern in *Bacillus subtilis*. Mol. Microbiol. 56, 1481–1494. 10.1111/j.1365-2958.2005.04659.x 15916600

[B44] Wang J. Zhang K. Xu L. Wang E. (2011). Quantifying the Waddington landscape and biological paths for development and differentiation. Proc. Natl. Acad. Sci. U.S.A. 108, 8257–8262. 10.1073/pnas.1017017108 21536909 PMC3100956

[B45] Zhang J. Tian X.-J. Zhang H. Teng Y. Li R. Bai F.. (2014). TGF-β –induced epithelial-to-mesenchymal transition proceeds through stepwise activation of multiple feedback loops. Sci. Signal. 7, ra91. 10.1126/scisignal.2005304 25270257

